# Gene expression of male pathway genes *sox9* and *amh* during early sex differentiation in a reptile departs from the classical amniote model

**DOI:** 10.1186/s12864-023-09334-0

**Published:** 2023-05-05

**Authors:** Susan Wagner, Sarah L. Whiteley, Meghan Castelli, Hardip R. Patel, Ira W. Deveson, James Blackburn, Clare E. Holleley, Jennifer A. Marshall Graves, Arthur Georges

**Affiliations:** 1 Institute for Applied Ecology, University of Canberra, Bruce, ACT Australia; 2 Australian National Wildlife Collection CSIRO, National Research Collections Australia, Crace, ACT Australia; 3 Genome Sciences Department. John Curtin School of Medical Research, Australian National University, Canberra, ACT Australia; 4 Kinghorn Centre for Clinical Genomics, Garvan Institute of Medical Research, Darlinghurst, NSW Australia; 5 Faculty of Medicine, St Vincent’s Clinical School, University of New South Wales, Sydney, NSW Australia; 6 School of Life Sciences, La Trobe University, Bundoora, VIC Australia

**Keywords:** Sex determination, Reptiles, *SOX9*, *AMH*, Gonad differentiation

## Abstract

**Background:**

Sex determination is the process whereby the bipotential embryonic gonads become committed to differentiate into testes or ovaries. In genetic sex determination (GSD), the sex determining trigger is encoded by a gene on the sex chromosomes, which activates a network of downstream genes; in mammals these include *SOX9*, *AMH* and *DMRT1* in the male pathway, and *FOXL2* in the female pathway. Although mammalian and avian GSD systems have been well studied, few data are available for reptilian GSD systems.

**Results:**

We conducted an unbiased transcriptome-wide analysis of gonad development throughout differentiation in central bearded dragon (*Pogona vitticeps*) embryos with GSD. We found that sex differentiation of transcriptomic profiles occurs at a very early stage, before the gonad consolidates as a body distinct from the gonad-kidney complex. The male pathway genes *dmrt1* and *amh* and the female pathway gene *foxl2* play a key role in early sex differentiation in *P. vitticeps*, but the central player of the mammalian male trajectory, *sox9*, is not differentially expressed in *P. vitticeps* at the bipotential stage. The most striking difference from GSD systems of other amniotes is the high expression of the male pathway genes *amh* and *sox9* in female gonads during development. We propose that a default male trajectory progresses if not repressed by a W-linked dominant gene that tips the balance of gene expression towards the female trajectory.

Further, weighted gene expression correlation network analysis revealed novel candidates for male and female sex differentiation.

**Conclusion:**

Our data reveal that interpretation of putative mechanisms of GSD in reptiles cannot solely depend on lessons drawn from mammals.

**Supplementary Information:**

The online version contains supplementary material available at 10.1186/s12864-023-09334-0.

## Background

Gonads are unusual in that two different organs, testis or ovary, develop from the same bipotential primordium, the genital ridge. In species with genetic sex determination (GSD), the fate of the bipotential gonad is determined at the point of conception by the complement of chromosomes inherited from the parents. Mammals usually have GSD with male heterogamety (XX females/XY males) whereby the presence or absence of the Y chromosome, and the gene *SRY* it harbours, determines the developmental trajectory leading to the sexual phenotype. *SRY* initiates the male regulatory pathway which leads to suppression of the female pathway. Without *SRY* the female regulatory pathway is pursued, which leads to suppression of the male pathway [1]. Birds also have GSD but with female heterogamety (ZW females/ZZ males). Here, dosage of *DMRT1*, a gene encoded and functional on the Z but not the W chromosome, determines whether the female or male regulatory pathway is taken [2, 3]. Only a two-fold difference in expression of *DMRT1* (expressed from two Z chromosomes in males *versus* only one Z in females) is sufficient to determine sexual fate. The two-fold expression difference occurs at a blastoderm stage before the mesonephros or genital ridges form, but in the gonadal lineage, expression of *dmrt1* is highly upregulated maintaining the two-fold dosage difference that determines sexual fate of the gonads [4]. In both birds and mammals, the sex determining gene (*DMRT1* or *SRY,* respectively) initiates the run-away upregulation of *SOX9,* a key transcriptional activator of male sex genes, a process required for testes differentiation [5]. With respect to sex determination, fishes are an interesting and the most diverse group, as all so far discovered vertebrate sex determination systems can be found among them [6].

Although the mammalian and avian GSD systems have been well studied, in reptiles no female or male GSD pathway has been investigated. Part of the reason is that, unlike mammals and birds, reptiles display a variety of sex determination systems, including genetic sex determination (GSD) and temperature sex determination (TSD) or a combination of the two. For instance, the central bearded dragon, *Pogona vitticeps,* has a ZZ male: ZW female system, but shows male to female sex reversal at elevated temperatures. Some reptiles with GSD have highly differentiated sex chromosomes (either male or female heterogamety), whereas others have poorly differentiated or homomorphic sex chromosomes [7]. Studies of the GSD turtles *Apalone spinifera* and *Apalone mutica* have been published but no female or male sex differentiation network has been described [8–11]. An efficient method to determine the sex chromosome complement in embryos is crucial for studying male and female GSD pathways, but was lacking for *A. spinifera* and *A. mutica* in the past [12]. Such sex chromosome PCR markers have been long established in *Pogona vitticeps* [13], the first reptile model species to have emerged for focused studies of male and female GSD pathways.

Most work on reptile sex determination pathways has concentrated on TSD species. In TSD the egg incubation temperature during the temperature sensitive window of development biases the percentage of male or female offspring [14]. In some species, the higher temperature leads to females, in others to males [15]. The environmental temperature signal is captured by ancient and ubiquitous signalling pathways in the cell that influence the epigenetic release or suppression of key sex genes [16, 17] to direct developmental programming of sex in the embryo [18, 19]. Downstream genes in the sex determining network of TSD species are similar to those acting in mammalian or avian GSD. For example, *dmrt1*, *sox9* and *amh* are involved in the male trajectory, *foxl2* and aromatase (*cyp19a1*) in the female trajectory [20–22].

Only few data are available on the genes in the sex determining network in GSD reptiles and no sex determining gene has been identified for any reptile, though there are candidates [23–25].

**Fig. 1:**
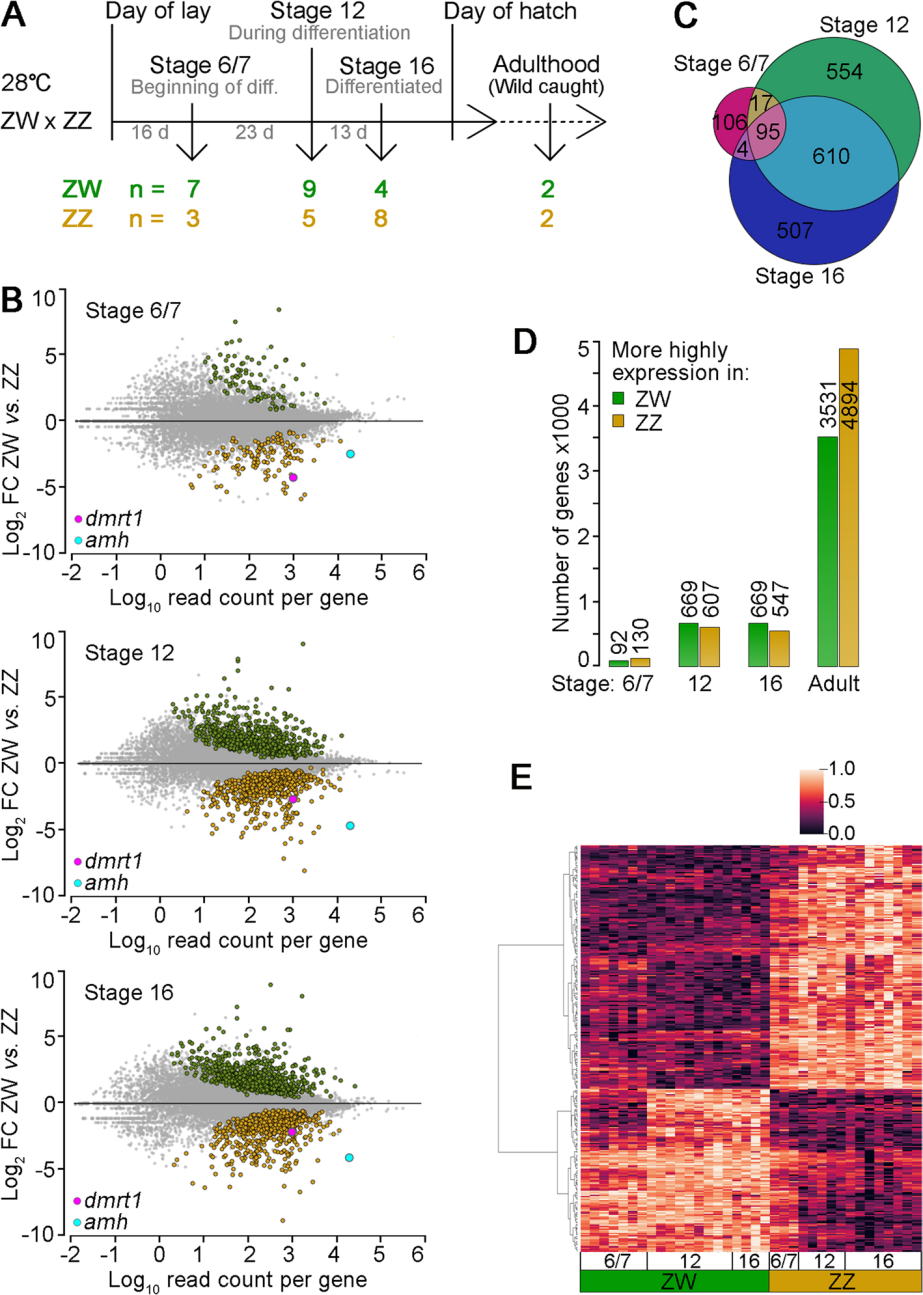
Male and female transcriptome profiles diverge early. **A** Experimental design with number of replicates per condition. **B** Log_2_ fold changes per gene of the entire detectable transcriptome are plotted against the normalised average read count per gene (log_10_). The comparison of female (ZW) *versus* male (ZZ) gonads is shown. Genes displaying a significantly different expression (adjusted *p*-value < 0.05) are coloured green if higher expressed in females or orange if higher expressed in males. FC: fold change. **C** Venn diagram showing the overlap of DEGs in female and male gonads between three stages. See Table S2 column J for respective gene lists. **D** Number of DEGs (adjusted *p*-value < 0.05) with a female bias (ZW, green) or male bias (ZZ, orange) in gonads at three developmental stages and in adults. See Table S2 column I for respective gene lists. **E** The normalised read count of the top 300 genes (lowest adjusted *p*-value) for all samples (columns) is presented as a heatmap. All values per gene (each row) were scaled between 0 and 1. Rows (genes) were clustered by the Ward variance minimization algorithm. The resulting dendrogram is shown on the left

We therefore set out to discover how gene expression profiles differ between male and female developmental trajectories under the control of the sex chromosomes in *Pogona vitticeps* and determine when in embryonic development sex differences first emerge. We compared expression profiles in the gonads of embryos of *Pogona vitticeps* incubated at moderate temperature (28 °C) at which no sex reversal occurs [13, 26, 27] (Fig. 1A). We demonstrate that sex specific differences have already emerged by the earliest stage at which we can dissect a consolidated gonad (stage 6 [28, 29]). Thus, sex determination under GSD occurs at a very early stage of gonadal differentiation. Patterns of expression of key genes in the vertebrate male sex determining network, *sox9* and *amh,* differ strikingly from those established in mammalian models. We found early engagement of *amh* in the developing testes. However, early sex-nonspecific expression of *sox9* leads us to propose that SOX9 is not a central hub for early reptile sex differentiation and there may be no positive feedback loop driving up the expression of SOX9 specifically in the male developmental trajectory of reptiles as it is of mammals and birds.

For the purpose of this paper, sex determination refers to the processes by which sexual fate is decided and sex differentiation refers to the regulatory processes that lead to development of either a testis or ovary. Sex determination in GSD species is most commonly associated with differential expression of genes encoded on sex chromosomes.

## Results

### Male and female gonad transcriptome profiles diverge at an early developmental stage

We compared gene expression of female and male gonads at three developmental stages, when gonads begin to differentiate (stage 6/7), are at an early stage of differentiation (stage 12) and are differentiated (stage 16), as well as in adults (Fig. 1A) [28, 29].

Female and male gonadal transcriptomes were distinguishable at the earliest stage at which we can dissect a consolidated gonad,stage 6/7 (Figure S1A); 222 genes were expressed differentially in male and female embryos at stage 6/7 (differentially expressed genes; DEGs; adjusted *p*-value < 0.05), with 130 of those genes showing a male bias and 92 showing a female bias (Fig. 1B, D; Table S2). As expected, over the course of ovary and testis development the number of DEGs increased (to *ca* 1250 at stage 12 and 16). Roughly the same proportion of genes was more highly expressed in ZZ or ZW developing gonads (Fig. 1B, D). As development progressed, the transcriptomic profiles of ZZ males and ZW females diverged further, as reflected by the increase in number of DEGs to 8,425 between adult ovaries and testes (Fig. 1D and S1B).

During embryonic development, genes typically had either a male or female bias; only 3 exchanged roles (*chrna1*, Cholinergic Receptor Nicotinic Alpha 1 Subunit; *noxo1*, NADPH oxidase organizer 1; and ENSPVIG00000017292, keratin type II cytoskeletal 5-like). A cluster heatmap of the 300 most relevant DEGs (lowest adjusted *p*-value) showed that one set of genes was upregulated in females and another set of genes was upregulated in males, each with a different degree of repression in the other sex (Fig. 1E). However, a few genes had the opposite bias during development and in adulthood (Figure S1E), presumably fulfilling different roles in the embryo and the adult.

Roughly half of the genes (52%) maintained their differential expression levels during embryonic development, once their expression had diverged (Fig. 1C and S1C, D and Table S2 columns J, K). However, 106 DEGs were specific for stage 6/7 and 554 DEGs were specific for stage 12 (Fig. 1C). Of the 106 DEGs that were dimorphic only at stage 6/7, 70 were more highly expressed in females and 36 were more highly expressed in males. In order to establish equal expression between males and females by stage 12, gene expression for most of those genes changed in female embryos adjusting to the expression in male embryos (Figure S2A). Those 106 stage 6/7 specific DEGs were not typically implicated with sex differentiation. The male biased group showed no gene ontology enrichment. However, the female biased group was enriched for gene ontology terms related to muscle development/contraction (Figure S2B). The 21 driver genes of that enrichment were much more highly expressed in female than in male gonads (4 to 64-fold), implying biological relevance. Expression of all but one of these genes was progressively reduced during female development until, by stage 12, they matched the low expression level of male gonads (Figure S2C-F). None of the 21 muscle related genes have been previously implicated in gonadal development or sex differentiation. It is difficult to see why muscle contraction should be implicated in ovary development, and the role of several of these genes in calcium regulation may be more pertinent, as calcium signalling is involved in sex reversal in *P. vitticeps* [18, 19]. Amongst the group are genes involved in Ca^2+^ signalling such as *myoz2* (Myozenin 2) which tethers calcineurin [30], *adrb2* (β-2-Adrenergic Receptor) which is associated with class C L-type calcium channel [31], subunits of troponin (*tnni1*, *tnni2*, *tnnc2*, *tnnt2*, *tnnt3*) which regulate muscle contraction via Ca^2+^ binding [32], and subunits of the nicotinic acetylcholine receptors (*chrnd* and *chrng*) which are non-selective cation channels [33]. It will be interesting to investigate if this group of genes is transiently expressed specifically in the female trajectory or if those genes are expressed in the precursor cell line, the intermediate mesoderm which develops into the gonads and kidneys. The switch in transcriptome reprogramming (i.e., the downregulation of redundant genes) might simply happen faster in the male trajectory.

### *Nr5a1* is involved in gonad development but probably not in sex differentiation

**Fig. 2:**
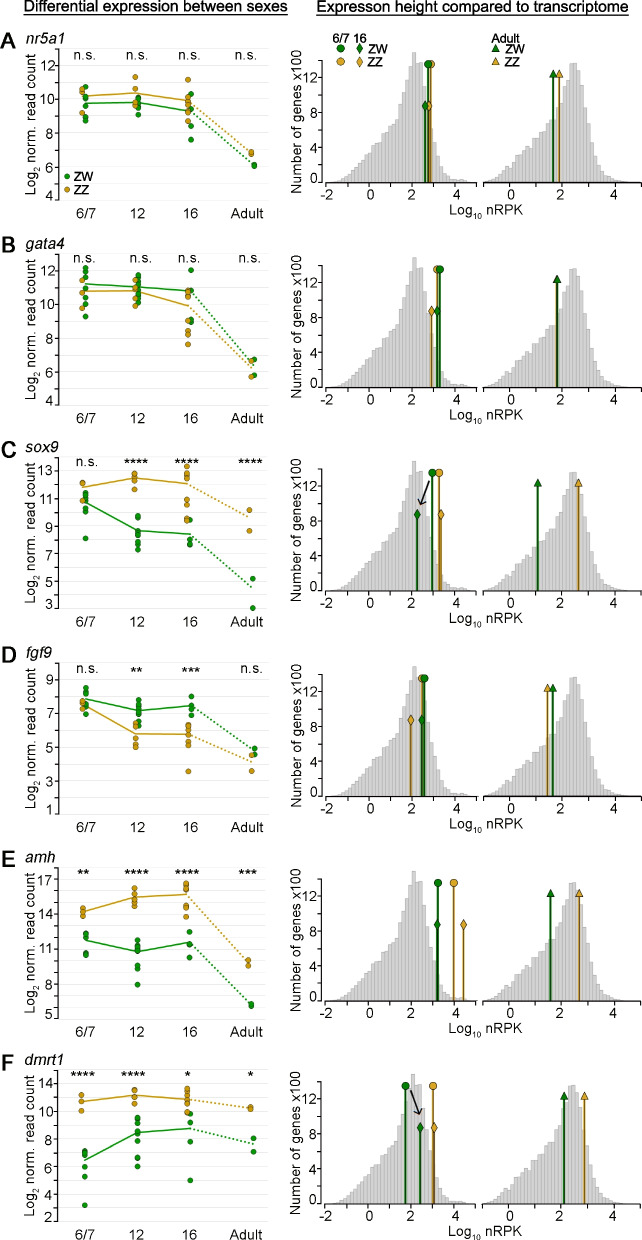
Differential expression of male sex differentiating genes. **A**—**F** Left panel: Log_2_ normalised read counts for six vertebrate sex-related genes (top of each panel) are plotted for all samples (green: ZW, orange: ZZ) over all stages (given at the bottom of the graph). The averages of all samples per sex and stage are presented as graphs, connected to the adult stage by dotted lines to acknowledge the much larger difference in time. The significance of the difference between ZW and ZZ is given for each stage on top of the graph as obtained from the differential expression analysis (adjusted *p*-value: * < 0.05, ** < 0.005, *** < 0.0005, **** < 0.00005, n.s. > 0.05). Middle and right panels: Histograms of log_10_ normalised read counts per gene (as obtained from the default normalisation of the DESeq2 R package) per kb transcript length (nRPK) for stage 6/7 (middle panel) and adults (right panel). The stage average nRPK for each gene (as in left panel) is indicated with a green or orange line for ZW or ZZ, respectively, for stage 6/7, stage 16 and for adults

Two genes that are highly expressed in undifferentiated gonads in both sexes of mammals and chicken are *nr5a1* (Nuclear Receptor Subfamily 5 Group A Member 1) and *gata4* (GATA Binding Protein 4) [2, 34, 35]. These marker genes were also highly expressed in *P. vitticeps* in both sexes from the earliest stage examined (Fig. 2A, B, right panels). Both genes continued to be highly, but not differentially, expressed, in both sexes through to stage 16. Adult gonads showed much lower expression of *nr5a1* and *gata4*, suggesting their main role is during embryonic development (Fig. 2A, B). Their similar expression patterns suggest a common role for *nr5a1* and *gata4* in the early formation of the genital ridge in amniotes.

*Nr5a1,* encoding the Steroidogenic Factor 1 (SF1), resides on the sex chromosomes of *P. vitticeps* [24]. Coupled with its role in sex determination in mammals, this location makes it a candidate for the master sex determining gene [24]. However, *nr5a1* showed no sex bias in expression levels that might be expected for a gene with a role in sex determination or differentiation.

Of the 302 protein coding genes (including *nr5a1*) and long non-coding RNA genes borne on one of the four sex chromosome scaffolds of *P. vitticeps* [24, 36], only one gene showed sex biased expression at stage 6/7, displaying a *ca* 2.6-fold higher expression in females (adj. *p*-value = 0.044). This was *ak8* (Adenylate Kinase 8). Adenylate kinases are small enzymes with a crucial role in energetic metabolism. They are nucleoside monophosphate kinases that catalyse the reversible transfer of the terminal phosphate group between nucleoside triphosphates and monophosphates (Figure S3A) [37]. Isoenzyme 8 is cytosolic and least studied of all isoforms [37]. As of now, it has not been involved in sex determination or differentiation. Another 29 sex chromosome genes (*ca* 10%) were differentially expressed between the sexes at later stages 12 and/or 16 (Figure S3A).

### Chromatin remodelling genes *jarid2* and* kdm6b* are not differentially expressed in GSD

Histone modifiers *jarid2* (Jumonji And AT-Rich Interaction Domain Containing 2) and *kdm6b* (Lysine Demethylase 6B) play a role in high temperature induced sex reversal in *P. vitticeps* [19, 38] and in other evolutionary disparate TSD species [16, 17, 20, 39–41]. Epigenetic regulation is also implicated in GSD sytems, for example in mice, where a Jumonji family member, *Jmjd1a* (Lysine Demethylase 3A), directly regulates expression of the sex determining gene *Sry* [42].

We found that during GSD in *P. vitticeps* both genes, *jarid2* and *kdm6b*, were highly expressed but showed no sex bias (Figure S4 A, B). Two transcript isoforms of *jarid2* and *kdm6b* are expressed in *P. vitticeps* ZZ and ZW embryonic gonads, but no significant difference was observed in their ratio between ZZ and ZW gonads (Whiteley et al. 2022, Science Advances).

Of 154 chromatin modifying genes (PANTHER protein class PC00077), only one was differentially expressed in *P. vitticeps* at embryonic stage 6/7, though at low abundance. *Samd7* (Sterile Alpha Motif Domain-Containing Protein 7) is a retina specific component of the chromatin modifier complex PRC1 [43] (Table S6). Only a few other chromatin modifying genes were differentially expressed between males and females at later embryonic stages, whereas roughly half of the chromatin modifying genes were differentially expressed between adult ovaries and testes (Table S6). This suggests that epigenetic regulation plays a major role in maintenance of differentiated adult gonads rather than in establishing differentiation during embryonic development.

### Known sex differentiation genes have unique expression profiles in *P. vitticeps*

**Fig. 3:**
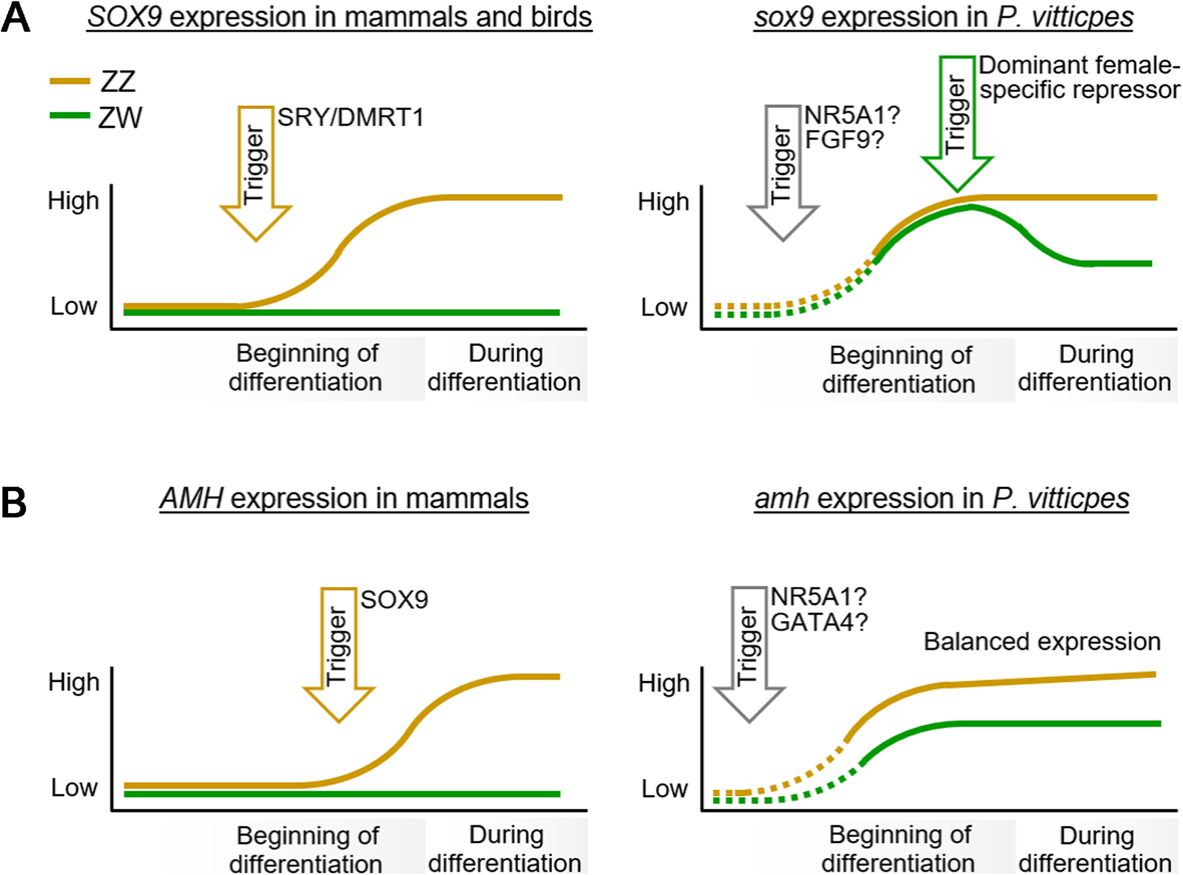
*sox9* and *amh* are highly expressed in female gonads in *P. vitticeps*. Schematic of expression in mammalian/chicken embryonic gonads for comparison (left panel) and in *P. vitticeps* as observed in this study (right panel). **A **sox9, **B** *amh. SOX9* expression in mammals or chicken is upregulated at the onset of gonad differentiation only in males (orange), while *sox9* expression was high already in both sexes in *P. vitticpes*. We assume *sox9* is not expressed in the precursor cell line and is upregulated early in the gonadal trajectory (dotted line). Once *sox9* expression reached its height in *P. vitticeps*, it was downregulated in females. *AMH* is usually not expressed in female mammals, while *amh* is highly expressed in both sexes in *P. vitticpes*, though with a strong male bias

Among transcription factors that were highly expressed in both sexes at stage 6/7 (Table S3) was *sox9* (SRY-Box Transcription Factor 9), a gene with a critical role in vertebrate sex determination. *Sox9* is autosomal in *P. vitticeps* [24, 36], implying that it is not the master sex determining gene in this species. We found that *sox9* expression was high in both sexes at stage 6/7. High expression is maintained in males through stage 12 and 16 but decreased with time in females (Fig. 2C). This pattern of expression of *sox9* in *P. vitticeps* is quite different from that in mammalian and avian systems, in which an initially low expression of *SOX9* is dramatically upregulated in males in response to differential expression of the sex determining gene [2, 44] (Fig. 3A).

*FGF9* (Fibroblast Growth Factor 9) and its receptor *FGFR2,* are highly expressed in male gonads and positively regulate *SOX9* expression during testis development in mammals [45, 46]. However, in *P. vitticeps* the expression of these genes showed no male bias at the beginning of differentiation, stage 6/7, and at stages 12 and 16 *fgf9* expression was higher in females than in males (Fig. 2D, S4C). Expression profiles of *sox9* and *fgf9* showed no correlation. In males *sox9* expression from stage 6/7 to stage 16 was constant while *fgf9* expression decreased. In females *fgf9* expression was constant while *sox9* expression decreased (Fig. 2C and S4A). Thus the positive feedback loop between *sox9* and *fgf9* in mammals seems not to operate in *P. vitticeps*, at least after stage 6/7. However, it remains possible that very early in development (prior to stage 6/7) *sox9* upregulation is supported by *fgf9* in both, male and female *P. vitticeps*, then later a dominant gene may suppress *sox9* expression in females despite constant levels of *fgf9* (Fig. 3A).

*AMH* (Anti-Müllerian Hormone) is differentially expressed early in mammalian sex determination, being upregulated by SOX9 in male gonads and absent in female [47]. We found that *amh* (ENSPVIG00000005953) is highly expressed in gonads at stage 6/7 in *P. vitticeps* females, and even more highly (about fourfold) expressed in males (Figs. 2E and 3B). Expression of *amh* remains constant in females, but in males increases to 16-fold over females during embryonic development. In adults of both sexes it is reduced, but still sexually dimorphic (Fig. 2E).

*Dmrt1* (Doublesex And Mab-3 Related Transcription Factor 1) is early acting in birds [2], as befits its role as the bird sex determining gene, but it has a later action in testis differentiation in mammals, especially mice [48]. In *P. vitticeps*, *dmrt1* is differentially expressed early in gonad development, showing a *ca* 19-fold higher expression in ZZ than in ZW gonads at stage 6/7, one of the strongest male biases early in development (Fig. 1B). In males, expression of *dmrt1* stayed high during the remainder of testis development through to stage 16. In females expression of *dmrt1* increased over time, from *ca* 19-fold lower expression than the male gonad to only *ca* fourfold lower expression by stage 16 and in adult ovaries (Figs. 1B and 2F). This expression profile strongly indicates an important role for *dmrt1* in early gonadal sex differentiation in *P. vitticeps* but it cannot be the master sex regulator due to its location on autosomal chromosome 2 and not the Z/W sex chromosomes [24].

**Fig. 4:**
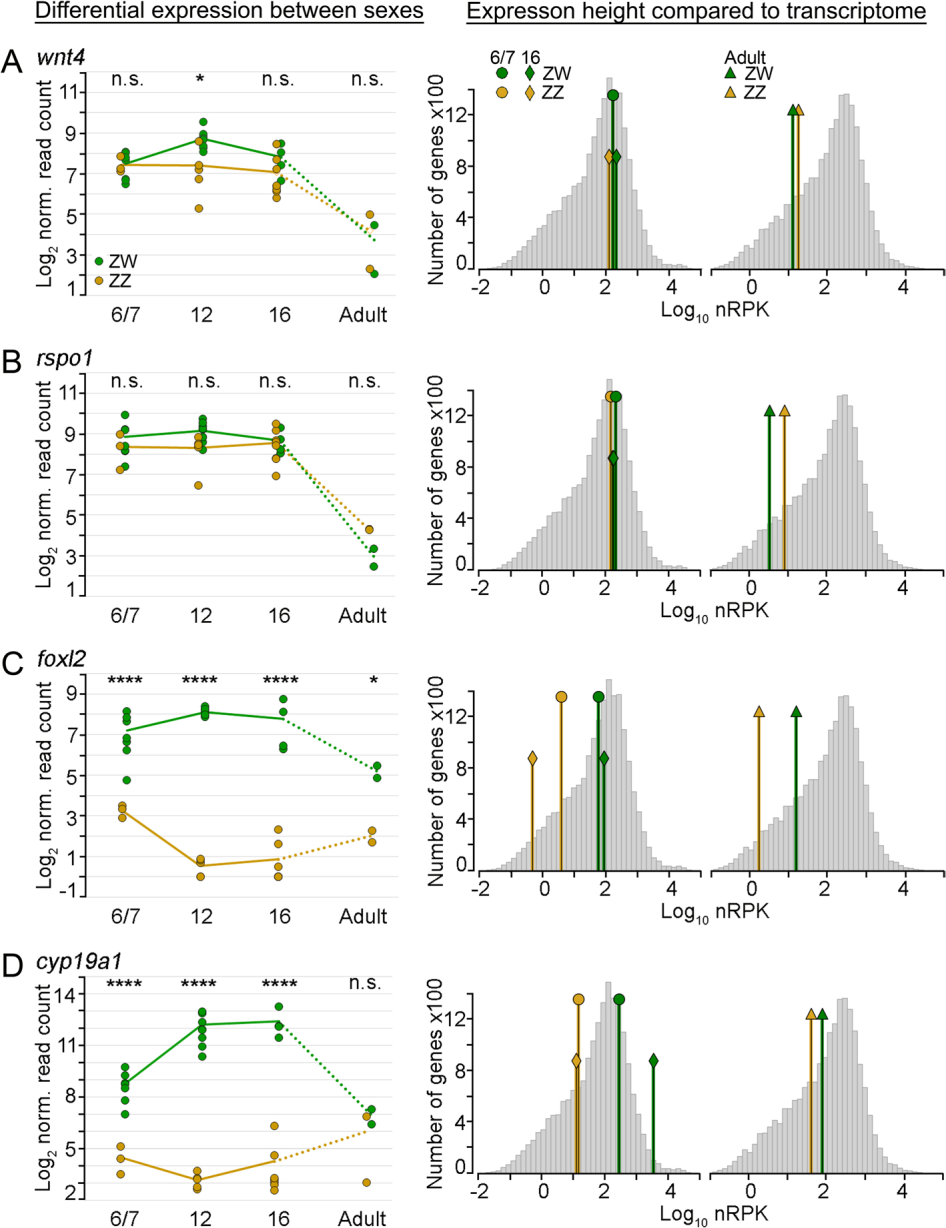
Differential expression of female sex differentiating genes. **A**—**D** Left panel: Log_2_ normalised read counts for six vertebrate sex-related genes (top of each panel) are plotted for all samples (green: ZW, orange: ZZ) over all stages (given at the bottom of the graph). The averages of all samples per sex and stage are presented as graphs, connected to the adult stage by dotted lines to acknowledge the much larger difference in time. The significance of the difference between ZW and ZZ is given for each stage on top of the graph as obtained from the differential expression analysis (adjusted *p*-value: * < 0.05, ** < 0.005, *** < 0.0005, **** < 0.00005, n.s. > 0.05). Middle and right panels: Histograms of log_10_ normalised read counts per gene (as obtained from the default normalisation of the DESeq2 R package) per kb transcript length (nRPK) for stage 6/7 (middle panel) and adults (right panel). The stage average nRPK for each gene (as in left panel) is indicated with a green or orange line for ZW or ZZ, respectively, for stage 6/7, stage 16 and for adults

In mammals, WNT signalling is active in the undifferentiated gonad, mediated by positive regulators *WNT4* (Wnt Family Member 4) and *RSPO1* (R-Spondin 1); after *SRY* activation in the male gonad, *WNT4* and *RSPO1* expression is downregulated to repress the female pathway [49–51]. In *P. vitticeps*, *wnt4* and *rspo1* were well expressed throughout embryonic development in both sexes; neither gene was downregulated in males (Fig. 4A, B). In fact, no positive regulators of WNT signalling (WNT ligands and frizzled receptors, including *wnt4* and *rspo1*) were differentially expressed at stage 6/7 (Table S4). Instead, five genes known to antagonize WNT signalling (*frzb*, *trabd2a* also known as *tiki1*, *shisa8*, *dkk3* and *wif1*) [52] all showed a male bias at stage 6/7 of gonad development (Figure S4D-H, Table S4). This suggests that control of WNT signalling in male *P. vitticeps* early in gonad differentiation is achieved by expression of WNT inhibitors rather than by suppression of WNT activators *wnt4* and *rspo1*. After the onset of differentiation, the expression of WNT pathway genes was more diverse. Several WNT ligands (*wnt2b*, *wnt4*, *wnt10a* and *wnt11*) displayed a female bias, while *wnt6* expression was male biased. Likewise, several WNT antagonists were male or female biased at stages 12 and/or 16 (Table S4).

The transcription factor FOXL2 (Forkhead Box L2) is expressed early and is female specific in mammalian gonads [53, 54]. In all female vertebrates FOXL2 is likely to be a key regulator of expression of *CYP19A1* (aromatase) [55], the enzyme controlling estrogen production [56]. Estrogen has a highly conserved role in nonmammalian vertebrates, but its function in the development of the mammalian ovary remains less clear [57]. Female markers *cyp19a1* and *foxl2* were more highly expressed in *P. vitticeps* females compared to males throughout all embryonic stages (Fig. 4C, D). Both genes were barely expressed in males. While *foxl2* expression in females had reached its height by stage 6/7 and was constant throughout development, expression of *cyp19a1* rose by roughly eightfold from stage 6/7 to 12, in line with the established view that *foxl2* regulates the expression of aromatase [55].

### Novel candidates for sex differentiation

Many other genes were dimorphically expressed throughout all embryonic stages; 17 with a female bias and 78 with a male bias (Table S5). Gene ontology terms enriched in both groups relate to sex development. Female specific terms included tube/vessel regulation and hormone secretion/metabolism. Male specific terms included regulation of meiosis, signalling, development and cell death (Figure S5A, B).

The five most highly ranked genes in the male biased group with at least 16-fold sex difference were *amh*, *col9a1* (Collagen Type IX Alpha 1 Chain, ENSPVIG00000019420), *nkx3-1* (NK3 Homeobox 1), *fmod* (Fibromodulin) and *spink2* (Serine Peptidase Inhibitor Kazal Type 2, ENSPVIG00000008173) (Table S5, Figure S5C-F). Of these genes only *amh* has a known role in embryonic gonad development [47]. NKX3-1 is a transcription factor expressed in the mammalian prostate and throughout its development, and is a well-established prostate tumour suppressor [58, 59]. A recent study demonstrated NKX3-1 expression in human Sertoli cells by immunohistology [60], although an earlier study detected only trace amounts of *NKX3-1* transcripts in human testes [61]. However, *Nkx3-1*^*−/−*^ knock-out rats displayed non-aberrant testis [62]. SPINK2 is a protease inhibitor that was shown to have a function in spermatogenesis but has not yet been reported as expressed during embryonic gonad development [63]. Based on their relevant expression pattern for male gonad differentiation in *P. vitticeps*, both genes are prime candidates for driving male gonad differentiation, in conjunction with established factors, such as *dmrt1* or *amh*. COL9A1 and FMOD are two extracellular matrix proteins. *Col9a1* was reported to be expressed with a male bias in the bipotential gonad of an amphibian, *Xenopus laevis* [64]. In mouse and chicken, the alpha 3 chain, *col9a3*, has been associated with male gonadal development, but it is unclear if COL9A3 is functioning alone or in a trimer together with the alpha chains 1 and 2 forming collagen type IX [65]. Likewise, the function of collagen type IX in male gonadogenesis is still unknown. The role of cell adhesion or the extracellular matrix (ECM) in establishing testicular or ovarian structure has not been well studied [66]. Our results from *P. vitticeps* suggest a likely role of ECM genes in early gonad differentiation and provide candidate genes for further studies.

The highest ranked gene in the female biased group was aromatase, *cyp19a1* (Table S5). To find more genes relevant for the female trajectory we performed a weighted correlation network analysis that yielded 25 modules (Figure S6A, Table S7). Two modules (module 8/pink and module 14/cyan) strongly correlated with female development (*p*-value < 0.01, correlation value > 0.7) (Figure S6B). Together they comprised 633 genes, amongst them aromatase *cyp19a1*, with the highest module-membership in module 14. Several signalling pathways were enriched in this gene set, MAPK, RAS, RAP1, PI3K-Akt, VEGF and calcium signalling (in ascending order of their false discovery rate) (Figure S6C). MAPK and PI3K-Akt pathways have been implicated in female gametogenesis in mammals [67] and it will be exciting to explore their influence in embryonic female gonad differentiation. Calcium signalling was suggested to be modulated by gonadal hormones [68] and in *P. vitticeps*, calcium signalling is thought to be important for the capture of the temperature cue and its transduction to cellular signalling pathways in male to female sex reversal [18, 19]. Our findings suggest that calcium signalling also plays a role in genetically determined female gonad development. VEGF and RAP1 signalling have not been implicated in ovary related functions, though RAP1 plays a role in mouse spermiogenesis [69]. RAP1 is a key regulator in cell junction formation [70] and VEGF signalling promotes endothelial cell growth, migration and survival, and plays a key role in angiogenesis [71]. The most significant enrichment was in genes involved in the pathway for axon guidance, which presumably have a different function in female gonad development, so might be moonlighting proteins [72].

Visualisation of the weighted gene correlation network helped to elucidate hub genes in female and male sex differentiation (Figure S7-9, Table S7). Two genes stood out as hub genes with strong positive membership in module 14/cyan (Figure S7A, Table S7), the module with the most significant and highest correlation with ZW genotype. One of them was aromatase *cyp19a1*. The other, transcription factor *lef1* (Lymphoid Enhancer Binding Factor 1), was shown to interact with ß-catenin to regulate target genes of WNT signalling [73]. It has not been strongly associated with gonadal sex differentiation so far. Hints come from two studies showing female-specific regulation of *LEF1* splicing in mouse gonadal transcriptomes [74], and downregulation of *lef1* in an ovary-to-testis gonadal transformation process in zebrafish [75]. Being a hub gene in female sex differentiation in *P. vitticeps*, *lef1* might function to orchestrate WNT signalling in female development. The most prominent hub gene in the second ZW module, 8/pink, was *hsd17b3* (Hydroxysteroid 17-Beta Dehydrogenase 3), a gene with a strong negative module-membership (Figure S8, Table S7). It is barely expressed in female gonads at any stage but highly expressed in male gonads. *Hsd17b3* has a well-known role in male sex differentiation as it catalyses the final step in the testosterone biosynthesis pathway [76]. Suppression of *hsd17b3* is important for female development [77]. We are providing with the unsigned correlation network a wealthy source for further investigation to better understand how *hsd17b3* is suppressed in female gonads. For example, two transcription factors that are connected with *hsd17b3* through a negative correlation were *tshz3* (Teashirt Zinc Finger Homeobox 3) and *lmx1a-like* (LIM Homeobox Transcription Factor 1 Alpha—Like, ENSPVIG00000024375), both, themselves prominent hub genes of module 8/pink with a positive module membership (Figure S8, Table S7). They were more highly expressed in female than male gonads throughout all stages (Table S2). *Tshz3* has been recently implicated into Müllerian duct formation in chicken [78] and *lmx1a* into ovary morphogenesis in the fruit fly [79].

Module 20/royalblue with significant correlation with ZW genotype (*p*-value = 0.006, correlation value = 0.45) comprised the earlier identified group of genes related to muscle development/contraction that was much more highly expressed in female than in male gonads at stage 6/7 and reduced expression until they matched the low expression level of male gonads by stage 12 (Figure S2B-F), This module formed a very concentric and balanced network (Figure S7B).

One module, 7/black, strongly correlated with male development (*p*-value < 0.01, correlation value > 0.7) (Figure S6B, S9, Table S7). The three most prominent hub genes, all with positive membership, were *met* (MET Proto-Oncogene, Receptor Tyrosine Kinase) and *tiam2* (TIAM Rac1 Associated GEF 2), which have not been associated with sex differentiation, and *plcb1* (Phospholipase C Beta 1) which was implicated recently in development of genitalia in male geese [80]. The next three most prominent hub genes were the transcription factor *nkx3-1* and the two extracellular matrix genes *fmod* and *col9a1*, three of the earlier mentioned genes with strong male bias in all three stages. They all constitute interesting and promising candidates for further exploration.

## Discussion

We undertook the first transcriptome wide analysis of embryonic gonad differentiation in a reptile with genetic sex determination. We found that gonad transcriptome profiles of ZZ male and ZW female embryos diverged early, being already differentiated at stage 6/7 [28, 29]. This suggests that determination of the male or female pathway in *P. vitticeps* happens before stage 6/7 and thus prior to the consolidation of the gonad into a distinct organ, and possibly before eggs are laid by the mother.

The master sex gene that initiates gonad differentiation is unknown in *P. vitticeps* and we hoped that it might be revealed by the differential expression of a gene on the sex chromosomes at an early stage. Based on our current understanding of other GSD systems, there are two possible mechanisms by which sex could be genetically determined in *P. vitticeps*: a female-dominance system similar to the male-dominance system in mammals, and a dosage dependent system similar to that in birds. These might be expected to yield different expression patterns. The mammalian male-dominant sex determiner, the Y-borne gene *SRY*, is expressed highly for only one day in mice (though more broadly in other mammals), and this is enough to initiate the male pathway [81, 82]. A dosage dependent system, such as the two-fold higher expression of *DMRT1* in male chickens [2], probably requires expression for longer during bipotential gonad development.

The strongest candidate sex determining gene identified so far in *P. vitticeps* is *nr5a1*, which has alleles on the Z and W sex chromosomes. However, we observed no differential expression at stage 6/7 of *nr5a1*, or other sex relevant genes on the sex chromosomes. It remains possible that our sampling missed a short burst of activity of a male- or female-dominant sex determiner before stage 6/7, or that subtle dosage shifts of a dosage-controlled gene were beyond the resolution of this study. Recently, we have described sex-specific transcript isoforms of *nr5a1* in gonads of adult *P. vitticeps*, which are likely to result in NR5A1 proteins with sex-specific function [83]. The technique of high-throughput short read sequencing used in this study is suboptimal to detect those isoforms as they differed in a GC rich and repetitive region. We cannot exclude the possibility that sex-specific isoforms or sex-specific post-transcriptional, translational or post-translational regulation of *nr5a1* or another sex chromosome-borne gene may influence sex determination. Genome-scale studies of the translational state to better understand post-transcriptional regulation of sex differentiation genes are needed.

There is also the possibility that other Z or W-borne genes will be discovered that have been missed from the *P. vitticeps* draft genome assembly [24, 36]*.* An improved chromosome scale genome assembly, ideally with phased sex chromosomes, may be required to identify the master sex gene in *P. vitticeps*.

*SOX9* is a key gene in mammalian and avian testis determination [5, 84, 85]. It is directly upregulated in males by the sex determining factor, SRY in mammals and DMRT1 in chicken [2]. *SOX9* is not upregulated in female mammals. As well as directly initiating *AMH* expression to effect Müllerian duct regression, SOX9 activates testis-differentiation genes and inhibits ß-catenin in the ovary differentiating pathway [5]. The expression profile of *sox9* in *P. vitticeps* suggests that SOX9 does not act as a central key player in early testis determination in this species, implying that it plays a different role than in mammals. We observed that* sox9* was highly expressed in female as well as male embryos at an early stage, then was later, after the onset of gonad differentiation, downregulated in females. Similar *sox9* expression was reported in embryonic gonads of the TSD turtle *T. scripta* [22, 86, 87]. Hence, in the absence of the necessary correlative and functional work on reptiles, interpretation of putative mechanisms of sex determination in reptiles cannot depend on lessons drawn from mammals.

Gonads of *T. scripta* initially develop morphologically like a testis, with primitive SOX9-positive sex cords that later degenerate at a female producing temperature when *sox9* expression is downregulated [22, 86]. We observed no similar development of cord-like structures in female *P. vitticeps* gonads [29]. The high initial *sox9* expression in *P. vitticeps* female embryos evidently does not masculinize female *P. vitticeps* embryos, suggesting either the action of a female specific inhibitor or that, in contrast to mammals, SOX9 in is not important in the regulatory pathways that dictate sexual fate in *P. vitticeps* or reptiles generally.

The function of a female-specific SOX9 inhibitor might be fulfilled by aromatase, an enzyme responsible for a key step in the biosynthesis of estrogens [88], which is expressed early and strongly in female *P. vitticeps* embryos. In a marsupial mammal and a human-derived testis cell line, estrogen can regulate gonadal development through the nucleocytoplasmic shuttling of SOX9. In particular, it can supress nuclear translocation of SOX9, so that SOX9 cannot fulfill its function as transcription activator of male pathway genes [89, 90]. A similar mechanism might prevent SOX9 in female *P. vitticeps* embryos from activating male pathway genes in the nucleus until after *sox9* expression decreases later in female embryonic development. Next to estrogen, also the signalling molecule prostaglandin D2 was shown to induce nuclear import of SOX9 in mammals which induces male gonad development [91–93]. We cannot draw any conclusion for *P. vitticeps*, as the gene for the prostaglandin synthase (L-Pgds or H-Pgds) has not yet been annotated in its genome. It will be exciting to explore the cellular location of SOX9 protein in embryonic gonads of *P. vitticeps*.

DMRT1 and SOX9 have been shown to functionally interact in mammals [94]. The lower expression level of *dmrt1* in female gonads of *P. vitticeps* at the early stage of development might prevent SOX9 from being fully functional.

The regulation of expression of *SOX9* transcripts differs in mammals and other vertebrates. In mammals, *SOX9* is activated by the Y chromosome borne *SRY* gene in male (but not female) embryos as an early event in testis determination. Then NR5A1, FGF9 and SOX9 itself establish and maintain high *SOX9* expression in male gonads. In chicken, DMRT1 is thought to initiate *SOX9* expression. A direct role for DMRT1 seems unlikely in *P. vitticeps* because *dmrt1* and *sox9* expression profiles were inversely correlated in females. Although, we cannot exclude that *dmrt1* and *sox9* are expressed in different cell types. Stronger candidates for *sox9* regulation in *P. vitticeps* are *nr5a1* and *fgf9,* which are expressed highly in both sexes and could be responsible for the initial early and high *sox9* expression in both sexes. The downregulation of *sox9* in *P. vitticeps* female embryos at later stages is unlikely the result from decreases in *nr5a1* and *fgf9* expression in females, since their expression levels do not change at later stages. Another possibility is a female specific dominant repressor of *sox9* expression. Two genes prominent in the female pathway in all vertebrates, *foxl2* and *cyp19a1*, are expressed more strongly in females than males at stage 6/7, and the sex differential of both is increased at later stages: *foxl2* by downregulation in male embryos at stage 12, and *cyp19a1* by upregulation in females. The identity of a female specific dominant repressor of *sox9* expression that acts to downregulate *sox9* after the bipotential stage in female embryos remains speculative.

In mammalian systems, expression of *AMH* and its receptor *AMHR* initiates the regression of Müllerian ducts in male embryos [95]. Post-natally AMH also has a role in controlling germ cell proliferation in both sexes [96, 97]. This latter role is seen also in fish and birds, and is thought to be the ancestral role of AMH in a vertebrate ancestor that lacked Müllerian ducts [98]. However, in several fish, a copy of *amh* or *amhr* has a primary role in sex determination and defines a Y chromosome, while autosomal *amh* and *amhr* are involved in germ cell proliferation. Not surprisingly, the regulation of *AMH* is different in mammals and fish [98]. In mammals it is induced by SOX9 activity, in combination with SF1 and GATA4 [99, 100]. In other systems, (chicken, *T. scripta,* and the American alligator *Alligator mississippiensis)* differential expression of *amh* was detected before differential expression of *sox9* [2, 22, 101], although its action might be delayed by post-transcriptional regulation [102]. *P. vitticeps* showed the non-mammalian pattern of early differential *amh* expression, which is high in both sexes but substantially higher in males. This suggests that in *P. vitticeps*, *amh* has a role in very early gonad differentiation as well as a later role in germ cell proliferation. *P. vitticeps amh* is not located on any of the four sex chromosome scaffolds identified in the *P. vitticeps* draft genome assembly [36]. In fact, the scaffold with *amh* is not amongst those that have been cytogenetically mapped [24]. An improved chromosome-scale genome assembly will determine whether or not *amh* is autosomal or encoded on the sex chromosomes.

The WNT/β-catenin signalling appears to be conserved among vertebrates as a key pathway in female gonad development that is repressed during male development, including fishes, reptiles with TSD, chickens and mammals. Commonly, WNT signalling activators *RSPO1* and *WNT4* finetune the strength of WNT signalling during sex differentiation being downregulated in males and/or upregulated in females [50, 103]. In *P. vitticeps* increased male expression of several WNT inhibitors is likely to result in the same outcome, lower protein levels of the effector β-catenin in males.

On the basis of our results, we propose a model for sex determination in *P. vitticeps* that is different to the mammalian system. In the classical model that was established in mammals, an upstream male determining factor initiates the runaway upregulation of *SOX9* in males, triggering expression changes in key sex differentiation genes (including *AMH*). This does not apply to *P. vitticeps*, and probably not to reptiles generally. Early and central players for male development seem to be *amh* and *dmrt1* rather than *SRY* and *SOX9* as seen in mammals. Higher *sox9* expression in *P. vitticeps* males is therefore a consequence, not a driver, of early sex differentiation. Nor does the model established for birds seem to apply, depending on *dmrt1* and *sox9* but not *amh*. Unlike in chicken, no known key male gene on the sex chromosomes showed a two-fold expression difference in gonads at the beginning of differentiation in *P. vitticeps*.

**Fig. 5:**
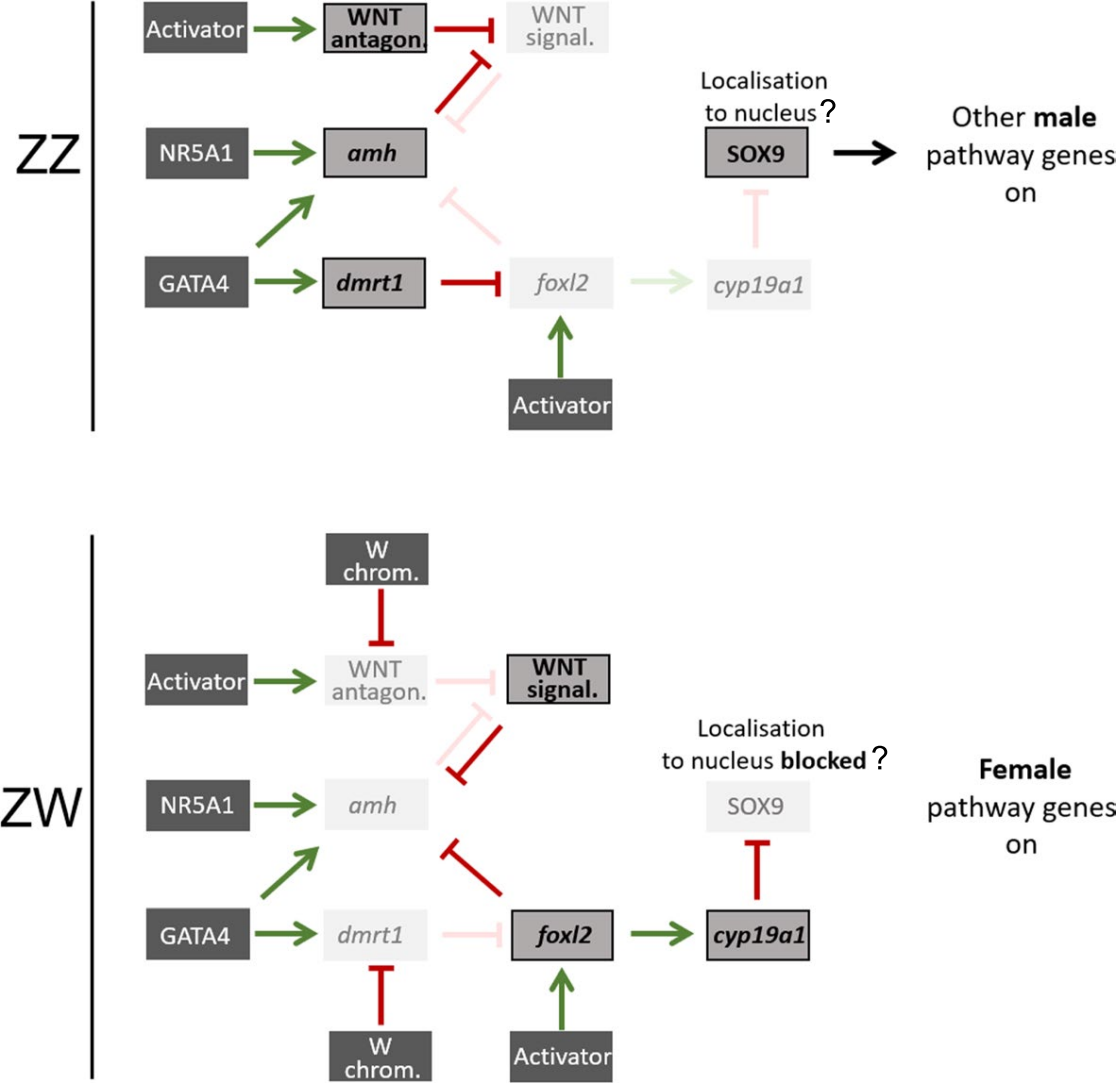
Model of early sex differentiation gene network. Temporal expression pattern of prominent driver genes of early sex differentiation in *P. vitticeps*. We propose a W-linked repressor of *dmrt1* and/or WNT-antagonists. We assume sex independent early activators (similar to the activator of *SRY* in mammals) of *dmrt1, amh,* WNT inhibitors and *foxl2* [54, 99, 100]. Interactions between genes are displayed by green arrows for a positive influence of expression and red blockage signs for a negative influence based on common and well-established relations in other species which our data did not oppose. In the absence of a W chromosome, *dmrt1* will eventually supress *foxl2* expression which leads to the establishment of extremely high *amh* expression levels in males. WNT signalling cannot be established, and the male trajectory can pursue. With a W chromosome present, *foxl2* expression can be established which in turn activates *cyp19a1* expression [55]. Further, WNT signalling will be strengthened owing to suppression of WNT inhibitors. Both, WNT signalling and *foxl2,* retain *amh* expression. High *cyp19a1* expression might subsequently block SOX9 nuclear localisation via estrogen. The balance of the expression network tips towards the female trajectory

This and the high expression of male genes *sox9* and *amh* in females after the onset of gonad differentiation led us to propose a dominance system for *P. vitticeps* in which the male trajectory is initially taken unless a dominant W-linked gene represses the male pathway to favour the female trajectory. In particular, expression of *amh*, *dmrt1*, WNT inhibitors, and *foxl2* might be activated at a certain stage in gonad development (Fig. 5), regardless of the sex chromosome complement. GATA4 was shown to activate *dmrt1* [104] and a sex-independent activator was proposed to activate *FOXL2* expression in mammals [54].

Which factor is responsible for the early and high expression of *amh in P. vitticeps*? In mammals it is SOX9, as well as SF1 and GATA4, amongst others [99, 100]. However, in some vertebrates *amh* expression preceded that of *sox9* [2, 22, 101]. In our study both genes were highly expressed at the earliest examined stage, especially *sox9,* which subsequently displayed a dimorphic pattern. We cannot exclude the possibility that SOX9, together with GATA4 and SF1, acts as a sex non-specific activator of *amh* expression early in gonad development. However, we cannot exclude early SOX9 inhibition in the female pathway by preventing nuclear localisation via action of estrogen. In the absence of the W chromosome, in ZZ males, *dmrt1* expression eventually supresses expression of *foxl2* and the male pathway continues (Fig. 5); and outcompetes the female pathway. In ZW females, a dominant W-linked gene, directly or indirectly, would inhibit *dmrt1* and WNT inhibitors, leading to establishment of WNT signalling and robust *foxl2* expression which would tip the balance towards the female trajectory (Fig. 5).

## Conclusions

Our study, the first RNA-sequencing of sex-typed GSD reptile embryos, provides a time series of embryonic gonad development in males and females. *P. vitticeps* shares downstream regulators of sex differentiation with reptile TSD species, and mammalian or avian GSD systems. However, those common regulators shift the timing of expression during gonad differentiation and the extent of sexually dimorphic expression. Expression profiles in *P. vitticeps* of key sex differentiation genes are more similar to known reptile TSD systems than they are to the mammalian GSD system. *P. vitticeps* has retained the central role for estrogen seen in lower vertebrates as high female specific expression of aromatase. However, *P. vitticeps* and possibly reptiles more broadly, seem to deviate from the mammalian expectation that early dimorphic upregulation of *sox9* is integral to early male differentiation, highlighting the potential for plasticity and novelty in reptilian GSD. The candidate sex differentiation genes identified here, broaden our perspective on vertebrate sex differentiation by introducing a cast of new players and roles. This work is an essential step towards the identification of elusive master sex determining gene/s that govern sexual fate in reptiles with sex chromosomes.

## Materials and methods

### Animal breeding, egg incubations and embryo sampling

Animal breeding, egg incubation and embryo sampling was performed as described in [19] and was in accordance with animal ethics protocols of the University of Canberra and ARRIVE guidelines (https://arriveguidelines.org/). Here repeated, eggs were obtained during the 2017–18 breeding season from the research breeding colony at the University of Canberra. Breeding groups comprised three females (ZW) to two males (ZZ). Paternity was confirmed by SNP genotyping [19]. Females were allowed to lay naturally, and eggs were collected at lay or within two hours of lay. Eggs were inspected for viability as indicated by presence of vasculature in the egg, and viable eggs were incubated at 28 °C in temperature-controlled incubators (± 1 °C) on damp vermiculite (4 parts water to 5 parts vermiculate by weight). Eggs were sampled at times corresponding to three developmental stages (6/7, 12 and 16). These stages equate to the beginning of gonad differentiation, recently differentiated gonad, and differentiated gonad, respectively, as described by Whiteley et al. [28, 29]. Between stages 4 to 8, gonads of both genotypes (ZW and ZZ) exhibit an elongated shape which develops into a rounder shape with defined cortex and medullary layers present at the end of this period and before gonad differentiation at stage 8/9. Testes differentiation is characterised by reduction of the cortex and proliferation of the medulla, within which seminiferous tubules form. Ovarian differentiation is characterised by a reduced medulla and a proliferating cortex with oogonia [29]. Sample sizes are given in Fig. 1A. Embryos were euthanized by intracranial injection of 0.1 ml sodium pentobarbitone (60 mg/ml in isotonic saline). Individual gonads were dissected from the mesonephros under a dissection microscope and snap frozen in liquid nitrogen. Isolation of the gonad from the surrounding mesonephros was considered essential for studying transcriptional profiles within the gonad. Embryos were genotyped using previously established protocols [13, 28]. Briefly, this involved obtaining a blood sample from the vasculature on the inside of the eggshell on an FTA® Elute micro card (Whatman). DNA was extracted from the card following the manufacturer protocols, and PCR was used to amplify a W specific region [13] so allowing the identification of ZW and ZZ samples.

### RNA extraction and sequencing

RNA extraction and sequencing was performed as described in [19]. Here repeated, RNA from isolated gonad samples was extracted in randomized batches using the Qiagen RNeasy Micro Kit (Cat. No. 74004) according to the manufacturer protocols. RNA was eluted in 14 μl of RNase free water and frozen at -80 °C prior to sequencing. Sequencing libraries were prepared in randomized batches using 50 ng RNA input and the Roche NimbleGen KAPA Stranded mRNA-Seq Kit (Cat. No. KK8420). All sample RNA and library DNA was quantified using a Qubit Instrument (ThermoFisher Scientific, Scoresby, Australia), with fragment size and quality assessment using a Bioanalyzer (Agilent Technologies, Mulgrave, Australia). Nine randomly selected samples were sequenced per lane using the Illumina HiSeq 2500 system at the Kinghorn Centre for Clinical Genomics (Garvan Institute of Medical Research, Sydney) in a paired-end mode with a read length of 150 bp and 25 million read-pairs per sample were obtained on average.

### Adult specimens

For the gonadal transcriptome analysis of wild caught adult *P. vitticeps* ZZ and ZW individuals we used a previously published dataset [36]. ENA accession numbers (https://www.ebi.ac.uk/ena) used for testes of ZZ male *P. vitticeps*: ERR753529, ERR413070; and for ovaries of ZW female *P. vitticeps*: ERR753530, ERR413082.

### Data analysis

The paired-end RNA-seq libraries for *P. vitticeps* were trimmed using cutadapt [105] with -q 20 -m 20 -max-n 4 -trim-n. Trimmed reads were aligned to the *P. vitticeps* genome assembly pvi1.1 (GCA_900067755.1; http://ftp.ensembl.org/pub/release-100/fasta/pogona_vitticeps/dna/Pogona_vitticeps.pvi1.1.dna.toplevel.fa.gz) [36] using STAR (v2.7.0f) [106] with splice-aware alignment guided by the accompanying Ensembl gene annotation (pvi1.1.100). Parameters were chosen to output unique alignments (–outFilterMultimapNmax 1). To reduce the number of scaffolds in the genome assembly, scaffolds with no genes annotated were excluded.

### Read count and differential gene expression analysis

Read count per gene was performed with htseq-count from the python package HTSeq [107] on the feature ‘exon’ (-t exon) with mode ‘union’ using the Ensembl gene annotation (pvi1.1.100) containing only genes tagged as protein-coding. Refer to Table S1 for raw counts per gene. In search for differentially expressed genes on the sex scaffolds, genes tagged as lncRNA (long non-coding RNA) were included as well (for Figure S3A). The R package DESeq2 (R 3.6.1, DESeq2 1.26.0 [108]) was used to perform differential gene expression analysis with default parameters. Genes with zero counts in all samples were excluded. Refer to Table S2 for complete results of a differential gene expression analysis between ZW and ZZ for each stage. To address gene expression changes from stage 6/7 to stage 12 differential gene expression analysis was performed between stages for each sex, ZZ and ZW. An adjusted *p*-value < 0.05 qualified the definition of differentially expressed gene (DEG) for a particular comparison. For normalisation the default method of DESeq2 was used, median of ratios, and whenever normalised read counts are plotted, this DESeq2 output was used. To compare expression levels of different genes the normalised read count was further normalised to the transcript length of the corresponding gene and expressed as nRPK (normalised read count per 1 kb transcript length). When more than one transcript was annotated for one gene, the average length of all annotated transcripts was used. If a gene of interest had no gene name assigned by the Ensembl gene annotation (pvi1.1.100), we looked for a gene name in the NCBI gene annotation (GCF_900067755.1 annotation release 100) by cross-referencing the Ensembl and NCBI gene IDs. Throughout the manuscript the Ensembl gene ID is given in addition to the gene name if a gene name was only assigned by the NCBI annotation and not by the Ensembl annotation. Such is the case for *amh* (ENSPVIG00000005953), *col9a1* (ENSPVIG00000019420) and *spink2* (ENSPVIG00000008173).

### Histograms of gene expression height

The average nRPK of all samples per stage was used to plot histograms of gene expression. The histogram of stage 6/7 and stage 16 is very similar (Figure S10). Therefore, expression height of individual genes for stage 6/7 and stage 16 was plotted on the background of the stage 6/7 histogram. While the histogram for adults is more different (for example most genes in adults have an nRPK of roughly 300, whereas in embryonic stages it is roughly 100), individual gene expression height for adults was plotted on the background of the adult histogram.

### Gene ontology analysis

Gene ontology enrichment analysis was done with ShinyGo v.0.66, http://bioinformatics.sdstate.edu/go/ [109], including inhere presented hierarchical clustering trees of gene ontology terms. As background served all protein-coding genes in the genome and the setting ‘Best matching species’ was used.

### Weighted correlation network analysis

The R package WGCNA (R 4.0.3, WGCNA 1.70–3 [110]) was used. Read counts per gene (genes tagged protein-coding and lncRNA) were used as input (see above). Only embryonic stages were included. Only genes with a count >  = 100 in at least two samples were included. For the gene network construction and identification of modules the following parameters were chosen: soft thresholding power = 10, type of network = unsigned (meaning that positive and negative correlations are considered), minimum module size = 20, merge cut height = 0.25. Figure panels S6D and E show the scale free topology model fit (signed R^2^) and the mean connectivity, respectively, over a range of soft thresholds.

For determination of the correlation between genotype and modules we have used the function ‘cor’ of the WGCNA package. To present positive correlations with the genotype ZZ, we have assigned ‘1’ for ZZ samples and ‘0’ for ZW samples (Figure S6B, left column). Vice versa, to present positive correlations with the genotype ZW, we have assigned ‘1’ for ZW samples and ‘0’ for ZZ samples (Figure S6B, right column). The two versions are mathematically redundant as they return same correlation values with opposite signs, and were chosen for presentational reason. Correlation values and *p*-values are given in Table S7.

For network visualisation Cytoscape (v. 3.9.1, [111]) was used. Visualizations are provided for modules that showed significant (*p* < 0.01) and high correlation (> 0.7) with ZZ or ZW genotype (Figures S7-9). The underlying nodes, edges and weights of those networks are provided in Table S7. Those modules were the 8/pink and 14/cyan having a strong correlation with ZW; and the 7/ black module having a strong correlation with ZZ. For meaningful visualisation only edges are represented with a weight > 0.08. Owing to the high number of nodes and edges in the black module, we provide a visualisation for edges with a weight > 0.11. The circular layout was chosen.

Gene nomenclature follows that of Kusumi et al. [112].

## Supplementary Information

**Additional file 1:** **FigureS1.** Globaldifferential gene expression analysis in gonadogenesis. **Figure S2.**Muscle related genes are female specific in gonads at the beginning of differentiation.**Figure S3.** Sex determining gene not anymore dimorphic at stage 6/7 oryet has not been mapped to sex chromosomes. **Figure S4.** Early inhibitionof WNT signalling in male gonads by expression of WNT inhibitors. **Figure S5.**Dimorphic genes throughout all embryonic stages - candidates for novel sexdifferentiation genes. **Figure S6.** Weighted correlation networkexpression to identify female related gene sets. **Figure S7.** Networkvisualisation to reveal hub genes of female sex differentiation. **Figure S8.**Network visualisation to reveal hub genes of female sex differentiation. **FigureS9.** Network visualisation to reveal hub genes of male sex differentiation. **FigureS10.** Histograms of gene expression height per stage for comparison.

**Additional file 2: Table S1.** Raw counts per gene for all gonadal transcriptomes obtained with RNA-seq.

**Additional file 3: Table S2.** Results of a differential gene expression analysis of ZW *versus *ZZ for each stage.

**Additional file 4 Table S3.** Relevant genes in gonad development, especially transcription factors.

**Additional file 5 Table S4.** Expression of genes involved in WNT signalling.

**Additional file 6 Table S5.** Dimorphic expressed genes throughout all three examined embryonic stages.

**Additional file 7 Table S6.** Expression of genes involved in chromatin remodelling.

**Additional file 8: Table S7.** Modules of genes resulting from a weighted correlation network analysis.

## Data Availability

Raw sequencing reads have been deposited in the Sequence Read Archive (SRA) repository of NCBI in BioProject PRJNA699086 (https://www.ncbi.nlm.nih.gov/bioproject/?term=PRJNA699086).
