# Supplementary material for: Gene expression of male pathway genes sox9 and amh during early sex differentiation in a reptile departs from the classical amniote model
Source: BMC Genomics. 2023 May 5;24:243. doi: 10.1186/s12864-023-09334-0 (PMC10163765; doi:10.1186/s12864-023-09334-0)
Supplement: Supplementary file 1 — Additional file 1: FigureS1. Globaldifferential gene expression analysis in gonadogenesis. Figure S2.Muscle related genes are female specific in gonads at the beginning of differentiation.Figure S3. Sex determining gene not anymore dimorphic at stage 6/7 oryet has not been mapped to sex chromosomes. Figure S4. Early inhibitionof WNT signalling in male gonads by expression of WNT inhibitors. Figure S5.Dimorphic genes throughout all embryonic stages - candidates for novel sexdifferentiation genes. Figure S6. Weighted correlation networkexpression to identify female related gene sets. Figure S7. Networkvisualisation to reveal hub genes of female sex differentiation. Figure S8.Network visualisation to reveal hub genes of female sex differentiation. FigureS9. Network visualisation to reveal hub genes of male sex differentiation. FigureS10. Histograms of gene expression height per stage for comparison. [file 12864_2023_9334_MOESM1_ESM.pdf]

SUPPLEMENTARY INFORMATION

Additional file 1: Figures S1 - S10.

Supplementary figure legends and supplementary figures (Figures S1 - S10).

Additional file 1: Figure S1 - S10.

FIGURE LEGENDS

Figure S1. Global differential gene expression analysis in gonadogenesis.

(A) The first two principal components of a read count per gene are shown for three embryonic stages and adults. All samples are plotted with female gonads in green (ZW) and male gonads in orange (ZZ).

(B) Log_2 fold changes per gene of the entire detectable transcriptome are plotted against the normalised average read count per gene (log_{10}) for adult ovaries (ZW) *versus* testes (ZZ). Genes displaying a significantly different expression (adjusted p-value < 0.05) are coloured green if higher expressed in females or orange if higher expressed in males. FC: fold change.

(C) Venn diagram showing the overlap of DEGs in female and male gonads between three embryonic stages and adults. For better orientation, numbers representing genes that established a sex differential expression during development and were differentially expressed also in adulthood are highlighted in red. Whereas, those that lost differential expression in adulthood are written in black. See Table S2 column K for respective gene lists.

(D) Legend for column K in Table S2. Genes were assigned a letter which indicates belonging to the corresponding groups in the Venn diagram shown in panel C.

(E) Some genes changed their sex bias between development and adulthood. Given is the number of DEGs with a sex bias at certain stages during development and how they divide in adulthood. The left and right boxes show DEGs with a female and male bias during development, respectively. Green circles: Female bias. Orange circles: Male bias. White circles: Not significantly differentially expressed genes (adjusted p-value > 0.05).

Figure S2. Muscle related genes are female specific in gonads at the beginning of differentiation.

(A) Expression changes from stage 6/7 to stage 12 of genes with a sex bias solely at stage 6/7. Schemes are illustrative for the type of change. * Significantly differentially expressed genes between stage 6/7 and 12 (adjusted p-value < 0.05). n.s. Not

significantly differentially expressed genes between stage 6/7 and 12 (adjusted p-value > 0.05).

(B) Hierarchical clustering tree of gene ontology terms in the category biological function enriched in the stage 6/7 specific set of genes with a female bias. The size of the blue dots corresponds to the level of significance and the false discovery rate is given next to the dot. For better readability related branches are marked by a green bar with a given summarising term.

(C) - (F) Expression profile of the muscle related gene set that was highly expressed in female gonads at stage 6/7 and had reduced expression to the low male level by stage 12. Average normalised read counts for three stages (given at the bottom of the graph) are plotted for each gene for females (green) and males (orange). The significance of the difference between stage 6/7 and stage 12 is given on top of the graph as obtained from the differential expression analysis, in green for females and in orange for males (adjusted p-value: * < 0.05, ** < 0.005, *** < 0.0005, **** < 0.00005, n.s. > 0.05). The genes were grouped according to the magnitude of their read count for better representation. In each graph, the genes are listed in order of their expression height in females at stage 6/7.

Figure S3. Sex determining gene not anymore dimorphic at stage 6/7 or yet has not been mapped to sex chromosomes.

(A) A list of 30 sex chromosome linked DEGs at any embryonic stage. Significant higher expression in ZW or ZZ is indicated in green or orange, respectively.

Figure S4. Early inhibition of WNT signalling in male gonads by expression of WNT inhibitors.

(A) - (H) Left panel: Log_2 normalised read counts for six vertebrate sex-related genes (top of each panel) are plotted for all samples (green: ZW, orange: ZZ) over all stages (given at the bottom of the graph). The averages of all samples per sex and stage are presented as graphs. The significance of the difference between ZW and ZZ is given for each stage on top of the graph as obtained from the differential expression analysis (adjusted p-value: * < 0.05, ** < 0.005, *** < 0.0005, **** < 0.00005, n.s. > 0.05). Right panels: Histograms of log_{10} normalised read counts per gene (as obtained from the

default normalisation of the DESeq2 R package) per kb transcript length (nRPK) for stage 6/7. The stage average nRPK for each gene (as in left panel) is indicated with a green or orange line for ZW or ZZ, respectively, for stage 6/7 and 16.

Figure S5. Dimorphic genes throughout all embryonic stages - candidates for novel sex differentiation genes.
(A) and (B) Hierarchical clustering tree of gene ontology terms in the category biological function enriched in the set of genes dimorphic expressed throughout embryonic development with a female bias (A) or male bias (B). The size of the blue dots corresponds to the level of significance and the false discovery rate is given next to the dot. For better readability related branches are marked by a rectangle with a given summarising term.
(C) - (F) Left panel: Log_2 normalised read counts for six vertebrate sex-related genes (top of each panel) are plotted for all samples (green: ZW, orange: ZZ) over all stages (given at the bottom of the graph). The averages of all samples per sex and stage are presented as graphs. The significance of the difference between ZW and ZZ is given for each stage on top of the graph as obtained from the differential expression analysis (adjusted p-value: * < 0.05, ** < 0.005, *** < 0.0005, **** < 0.00005, n.s. > 0.05). Right panels: Histograms of log_{10} normalised read counts per gene (as obtained from the default normalisation of the DESeq2 R package) per kb transcript length (nRPK) for stage 6/7. The stage average nRPK for each gene (as in left panel) is indicated with a green or orange line for ZW or ZZ, respectively, for stage 6/7 and 16.

Figure S6. Weighted correlation network expression to identify female related gene sets.
(A) Clustering dendrogram of genes, with dissimilarity based on topological overlap, together with assigned module colours.
(B) Associations between traits and modules. Each row corresponds to a module eigengene, column to a trait (genotype ZZ or ZW). The table is color-coded by correlation according to the colour legend. Modules 8/pink and 14/cyan showed strong correlation (p-value < 0.01, correlation value > 0.7) with the female trait, module 7/black with the male trait.

(C) Hierarchical clustering tree of Kyoto Encyclopedia of Genes and Genomes (KEGG) pathways enriched in modules 8/pink and 14/cyan. The size of the blue dots corresponds to the level of significance and the false discovery rate is given next to the dot.
(D) and (E) Analysis of network topology for various soft thresholding powers: scale free topology model fit (D) and mean connectivity (E). The soft thresholding power 10 was chosen for the network construction. The red horizontal line in (D) indicates y=0.9.

Figure S7. Network visualisation to reveal hub genes of female sex differentiation. Network visualisation of different modules. Edges with a weight > 0.08 are displayed. Genes with positive or negative membership are shown in light blue or grey, respectively. Genes without annotated gene name are displayed by the last 5 digits of their Ensembl gene ID ENSPVIG000000xxxxx.
(A) Module 14/cyan. The two most prominent hub genes are highlighted and were pulled out of the circle for better visibility.
(B) Module 20/royalblue. This module comprises the female and stage 6/7 specific group of genes which is related to muscle development/contraction (indicated in purple). All members of this module have positive membership.

Figure S8. Network visualisation to reveal hub genes of female sex differentiation. Network visualisation of module 8/pink. Edges with a weight > 0.08 are displayed. Genes with positive or negative membership are shown in light blue or grey, respectively. Genes without annotated gene name are displayed by the last 5 digits of their Ensembl gene ID ENSPVIG000000xxxxx. Prominent hub genes are highlighted and were pulled out of the circle for better visibility. ENSPVIG00000024375 is LIM homeobox transcription factor 1-alpha-like.

Figure S9. Network visualisation to reveal hub genes of male sex differentiation. Network visualisation of module 7/black. Edges with a weight > 0.11 are displayed. Genes with a positive or negative membership are shown in light blue or grey, respectively. Genes without annotated gene name are displayed by the last 5 digits of their Ensembl gene ID ENSPVIG000000xxxxx. The six most prominent hub genes, genes

with the most connections, are highlighted and were pulled out of the circle for better visibility.

Figure S10. Histograms of gene expression height per stage for comparison.
Histograms of log_{10} normalised read counts per gene (as obtained from the default normalisation of the DESeq2 R package) per kb transcript length (nRPK). The stage average nRPK per gene is presented. All three embryonic stages have very similar histograms while the histogram for adults is more different.
(A) Stage 6/7, (B) Stage 12, (C) Stage 16, (D) Adults.

Figure S1

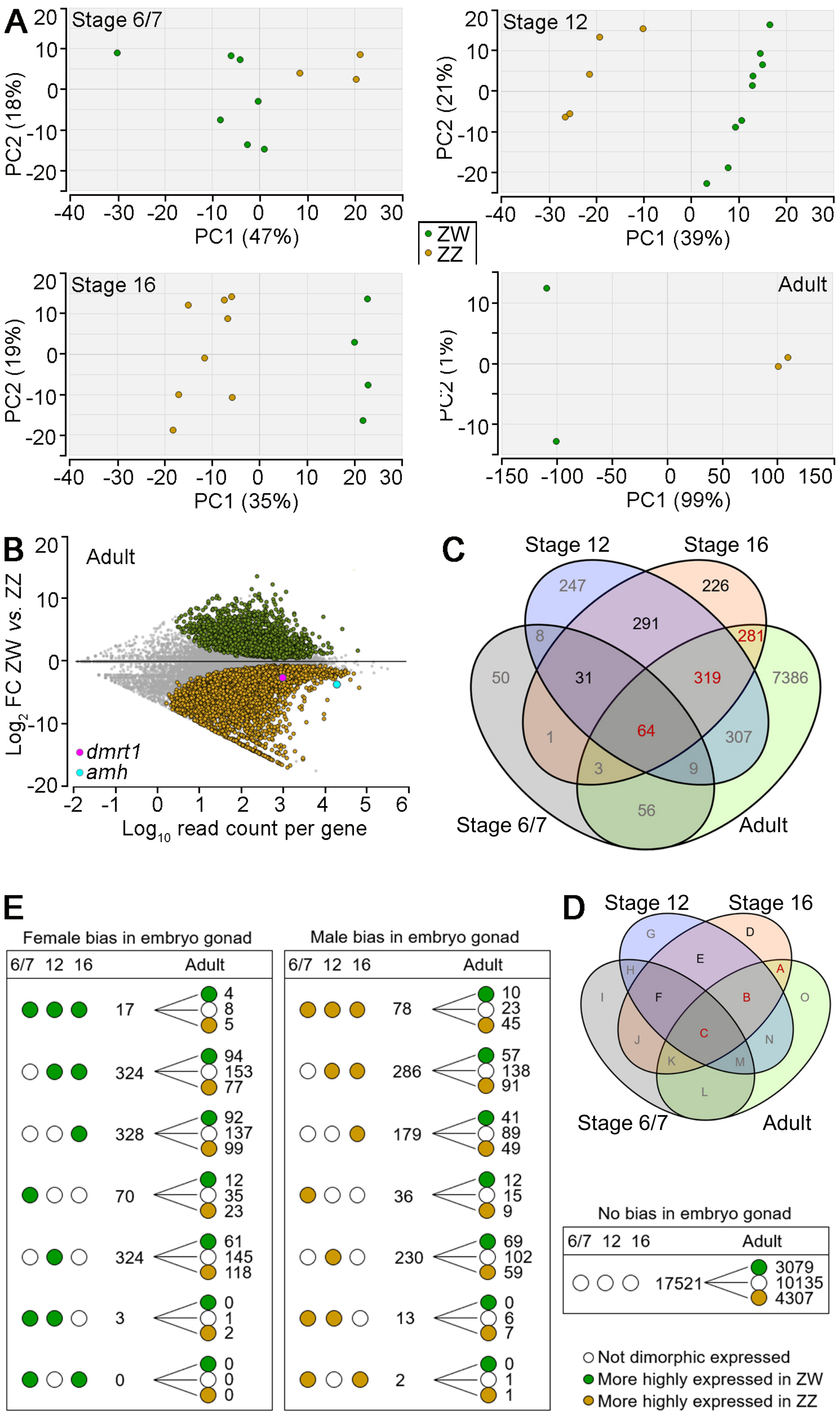

A
Stage 6/7
PC2 (18%)
PC1 (47%)
Stage 12
PC2 (21%)
PC1 (39%)
ZW
ZZ
Stage 16
PC2 (19%)
PC1 (35%)
Adult
PC1 (99%)
B
Adult
Log2 FC ZW vs. ZZ
dmrt1
amh
Log10 read count per gene
C
Stage 12
Stage 16
Stage 6/7
Adult
247
226
291
8
281
50
31
319
7386
1
64
307
3
9
56
E
Female bias in embryo gonad
6/7 12 16 Adult
17 4 8 5
324 94 153 77
328 92 137 99
70 12 35 23
324 61 145 118
3 0 1 2
0 0 0 0
Male bias in embryo gonad
6/7 12 16 Adult
78 10 23 45
286 57 138 91
179 41 89 49
36 12 15 9
230 69 102 59
13 0 6 7
2 0 1 1
D
Stage 12
Stage 16
Stage 6/7
Adult
G D E H A I F B O J C N K M L
No bias in embryo gonad
6/7 12 16 Adult
17521 3079 10135 4307
Not dimorphic expressed
More highly expressed in ZW
More highly expressed in ZZ

Figure S2

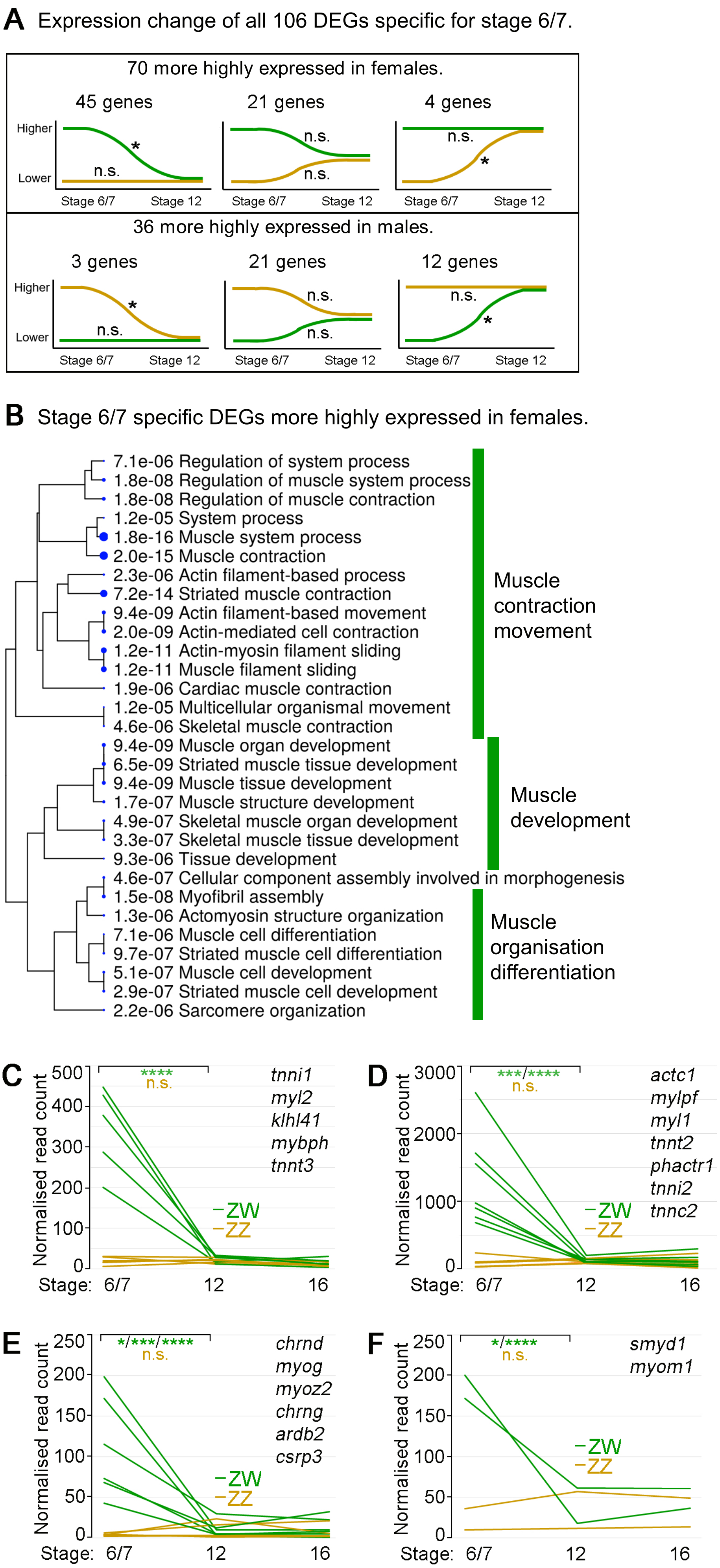
A Expression change of all 106 DEGs specific for stage 6/7.
70 more highly expressed in females.
45 genes
21 genes
4 genes
Higher
Lower
n.s.
*
Stage 6/7
Stage 12
36 more highly expressed in males.
3 genes
21 genes
12 genes
B Stage 6/7 specific DEGs more highly expressed in females.
7.1e-06 Regulation of system process
1.8e-08 Regulation of muscle system process
1.8e-08 Regulation of muscle contraction
1.2e-05 System process
1.8e-16 Muscle system process
2.0e-15 Muscle contraction
2.3e-06 Actin filament-based process
7.2e-14 Striated muscle contraction
9.4e-09 Actin filament-based movement
2.0e-09 Actin-mediated cell contraction
1.2e-11 Actin-myosin filament sliding
1.2e-11 Muscle filament sliding
1.9e-06 Cardiac muscle contraction
1.2e-05 Multicellular organismal movement
4.6e-06 Skeletal muscle contraction
9.4e-09 Muscle organ development
6.5e-09 Striated muscle tissue development
9.4e-09 Muscle tissue development
1.7e-07 Muscle structure development
4.9e-07 Skeletal muscle organ development
3.3e-07 Skeletal muscle tissue development
9.3e-06 Tissue development
4.6e-07 Cellular component assembly involved in morphogenesis
1.5e-08 Myofibril assembly
1.3e-06 Actomyosin structure organization
7.1e-06 Muscle cell differentiation
9.7e-07 Striated muscle cell differentiation
5.1e-07 Muscle cell development
2.9e-07 Striated muscle cell development
2.2e-06 Sarcomere organization
Muscle contraction movement
Muscle development
Muscle organisation differentiation
C
Normalised read count
500
400
300
200
100
0
****
n.s.
tnni1
myl2
klhl41
mybph
tnnt3
ZW
ZZ
Stage: 6/7 12 16
D
3000
2000
1000
0
/*
n.s.
actc1
mylpf
myl1
tnnt2
phactr1
tnni2
tnnc2
ZW
ZZ
Stage: 6/7 12 16
E
250
200
150
100
50
0
*/***/****
n.s.
chrnd
myog
myoz2
chrng
ardb2
csrp3
ZW
ZZ
Stage: 6/7 12 16
F
250
200
150
100
50
0
*/****
n.s.
smyd1
myom1
ZW
ZZ
Stage: 6/7 12 16

Figure S3

A

Gene ID	Gene name	Stage 6/7	Stage 12	Stage 16	Gene description
ENSPVIG00000014108	AK8	ZW	-	-	adenylate kinase 8
ENSPVIG00000013118		-	ZW	-	lipocalin-15-like LOC110087465 (cross-reference to NCBI)
ENSPVIG00000015873		-	ZW	-	formin binding protein 1 FNBP1 (cross-reference to NCBI)
ENSPVIG00000019061	EGFL7	-	ZW	-	EGF like domain multiple 7
ENSPVIG00000025698	PTGS1	-	ZW	-	prostaglandin-endoperoxide synthase 1
ENSPVIG00000025714	ADGRD2	-	ZW	-	adhesion G protein-coupled receptor D2
ENSPVIG00000015889	ZDHHC12	-	ZW	ZW	zinc finger DHHC-type containing 12
ENSPVIG00000018140	DNLZ	-	ZW	ZW	DNL-type zinc finger
ENSPVIG00000019070	OLFM1	-	ZW	ZW	olfactomedin 1
ENSPVIG00000021223		-	ZW	ZW	PBX homeobox 3
ENSPVIG00000021237		-	ZW	ZW	Novel gene
ENSPVIG00000025676	NPDC1	-	ZW	ZW	neural proliferation, differentiation and control 1
ENSPVIG00000025711	LHX2	-	ZW	ZW	LIM homeobox 2
ENSPVIG00000002419	SH2D3C	-	-	ZW	SH2 domain containing 3C
ENSPVIG00000013078	RNF208	-	-	ZW	ring finger protein 208
ENSPVIG00000015946	ENDOG	-	-	ZW	endonuclease G
ENSPVIG00000015765	FUBP3	-	ZZ	-	far upstream element binding protein 3
ENSPVIG00000018090	CRAT	-	ZZ	-	carnitine O-acetyltransferase
ENSPVIG00000021234	RALGPS1	-	ZZ	-	Ral GEF with PH domain and SH3 binding motif 1
ENSPVIG00000015755	AIF1L	-	ZZ	ZZ	allograft inflammatory factor 1 like
ENSPVIG00000015756		-	ZZ	ZZ	Novel gene
ENSPVIG00000018127	NACC2	-	ZZ	ZZ	NACC family member 2
ENSPVIG00000018136		-	ZZ	ZZ	G protein signaling modulator 1 GPSM1 (cross-reference to NCBI)
ENSPVIG00000018137		-	ZZ	ZZ	G protein signaling modulator 1 GPSM1 (cross-reference to NCBI)
ENSPVIG00000018188	NOTCH1	-	ZZ	ZZ	notch receptor 1
ENSPVIG00000021242	GARNL3	-	ZZ	ZZ	GTPase activating Rap/RanGAP domain like 3
ENSPVIG00000021248	GSN	-	ZZ	ZZ	gelsolin
ENSPVIG00000013119		-	-	ZZ	lipocalin-15-like
ENSPVIG00000013383		-	-	ZZ	ral guanine nucleotide dissociation stimulator
ENSPVIG00000018022	MIGA2	-	-	ZZ	mitoguardin 2

Figure S4

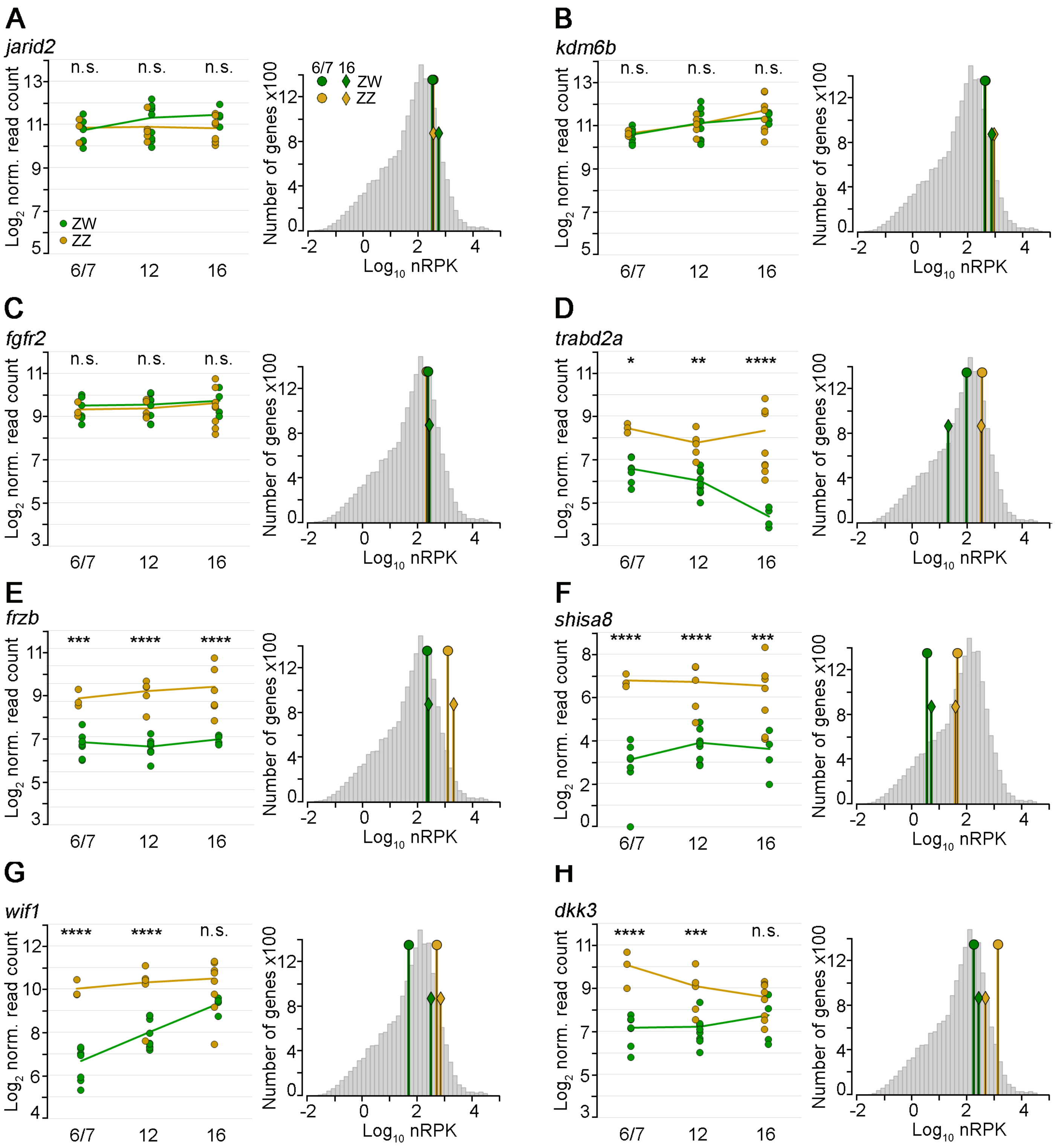

A
jarid2
n.s.
n.s.
n.s.
ZW
ZZ
6/7
12
16
Log2 norm. read count
6/7 16
ZW
ZZ
Number of genes x100
Log10 nRPK
B
kdm6b
n.s.
n.s.
n.s.
C
fgfr2
n.s.
n.s.
n.s.
D
trabd2a
*
**
****
E
frzb
***
****
****
F
shisa8
****
****
***
G
wif1
****
****
n.s.
H
dkk3
****
***
n.s.

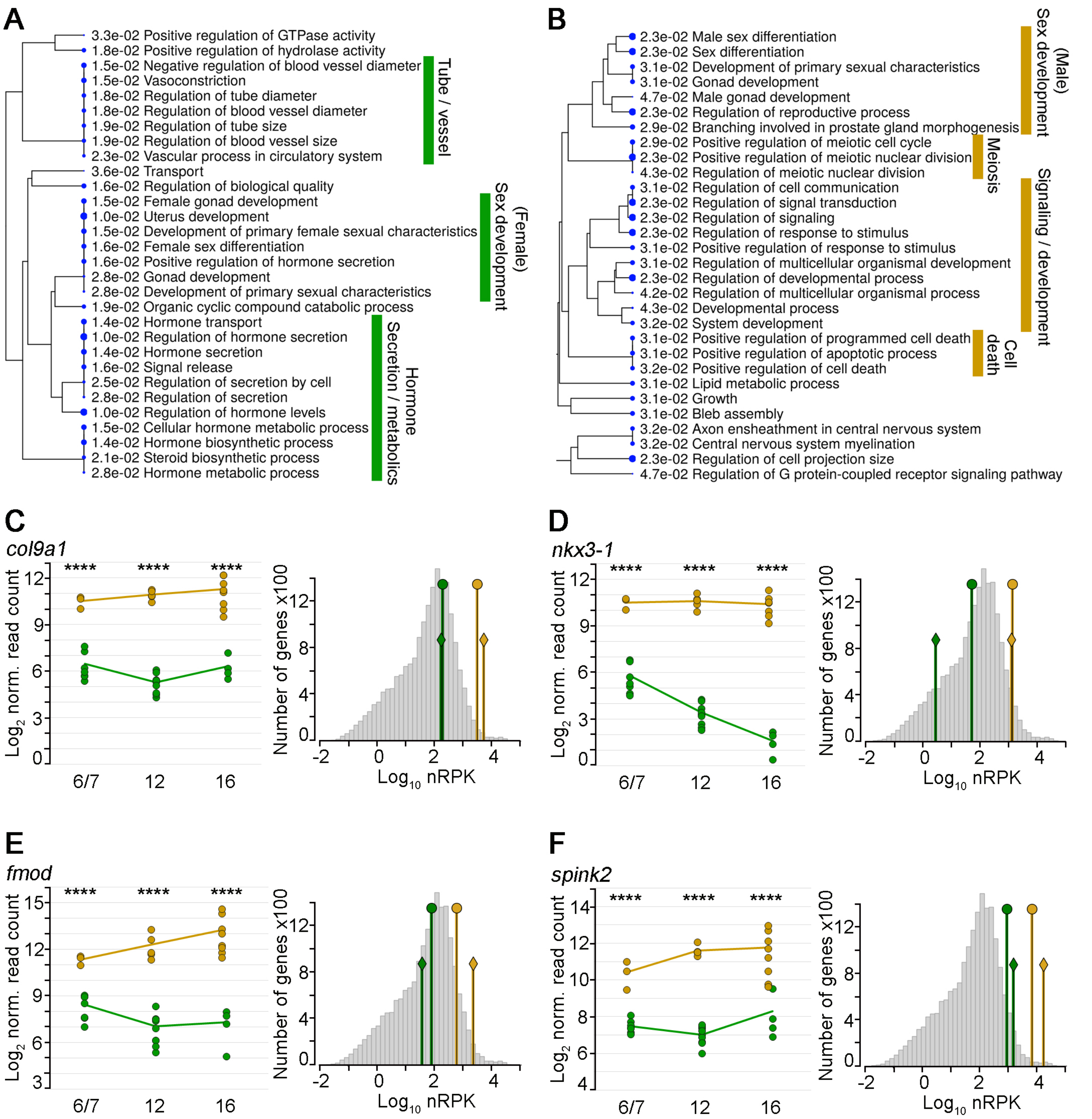
A
3.3e-02 Positive regulation of GTPase activity
1.8e-02 Positive regulation of hydrolase activity
1.5e-02 Negative regulation of blood vessel diameter
1.5e-02 Vasoconstriction
1.8e-02 Regulation of tube diameter
1.8e-02 Regulation of blood vessel diameter
1.9e-02 Regulation of tube size
1.9e-02 Regulation of blood vessel size
2.3e-02 Vascular process in circulatory system
3.6e-02 Transport
1.6e-02 Regulation of biological quality
1.5e-02 Female gonad development
1.0e-02 Uterus development
1.5e-02 Development of primary female sexual characteristics
1.6e-02 Female sex differentiation
1.6e-02 Positive regulation of hormone secretion
2.8e-02 Gonad development
2.8e-02 Development of primary sexual characteristics
1.9e-02 Organic cyclic compound catabolic process
1.4e-02 Hormone transport
1.0e-02 Regulation of hormone secretion
1.4e-02 Hormone secretion
1.6e-02 Signal release
2.5e-02 Regulation of secretion by cell
2.8e-02 Regulation of secretion
1.0e-02 Regulation of hormone levels
1.5e-02 Cellular hormone metabolic process
1.4e-02 Hormone biosynthetic process
2.1e-02 Steroid biosynthetic process
2.8e-02 Hormone metabolic process
Tube / vessel
(Female) Sex development
Hormone Secretion / metabolics
B
2.3e-02 Male sex differentiation
2.3e-02 Sex differentiation
3.1e-02 Development of primary sexual characteristics
3.1e-02 Gonad development
4.7e-02 Male gonad development
2.3e-02 Regulation of reproductive process
2.9e-02 Branching involved in prostate gland morphogenesis
2.9e-02 Positive regulation of meiotic cell cycle
2.3e-02 Positive regulation of meiotic nuclear division
4.3e-02 Regulation of meiotic nuclear division
3.1e-02 Regulation of cell communication
2.3e-02 Regulation of signal transduction
2.3e-02 Regulation of signaling
2.3e-02 Regulation of response to stimulus
3.1e-02 Positive regulation of response to stimulus
3.1e-02 Regulation of multicellular organismal development
2.3e-02 Regulation of developmental process
4.2e-02 Regulation of multicellular organismal process
4.3e-02 Developmental process
3.2e-02 System development
3.1e-02 Positive regulation of programmed cell death
3.1e-02 Positive regulation of apoptotic process
3.2e-02 Positive regulation of cell death
3.1e-02 Lipid metabolic process
3.1e-02 Growth
3.1e-02 Bleb assembly
3.2e-02 Axon ensheathment in central nervous system
3.2e-02 Central nervous system myelination
2.3e-02 Regulation of cell projection size
4.7e-02 Regulation of G protein-coupled receptor signaling pathway
(Male) Sex development
Meiosis
Signaling / development
Cell death
C
col9a1
D
nkx3-1
E
fmod
F
spink2
Log2 norm. read count
****
6/7
12
16
Number of genes x100
Log10 nRPK

Figure S6

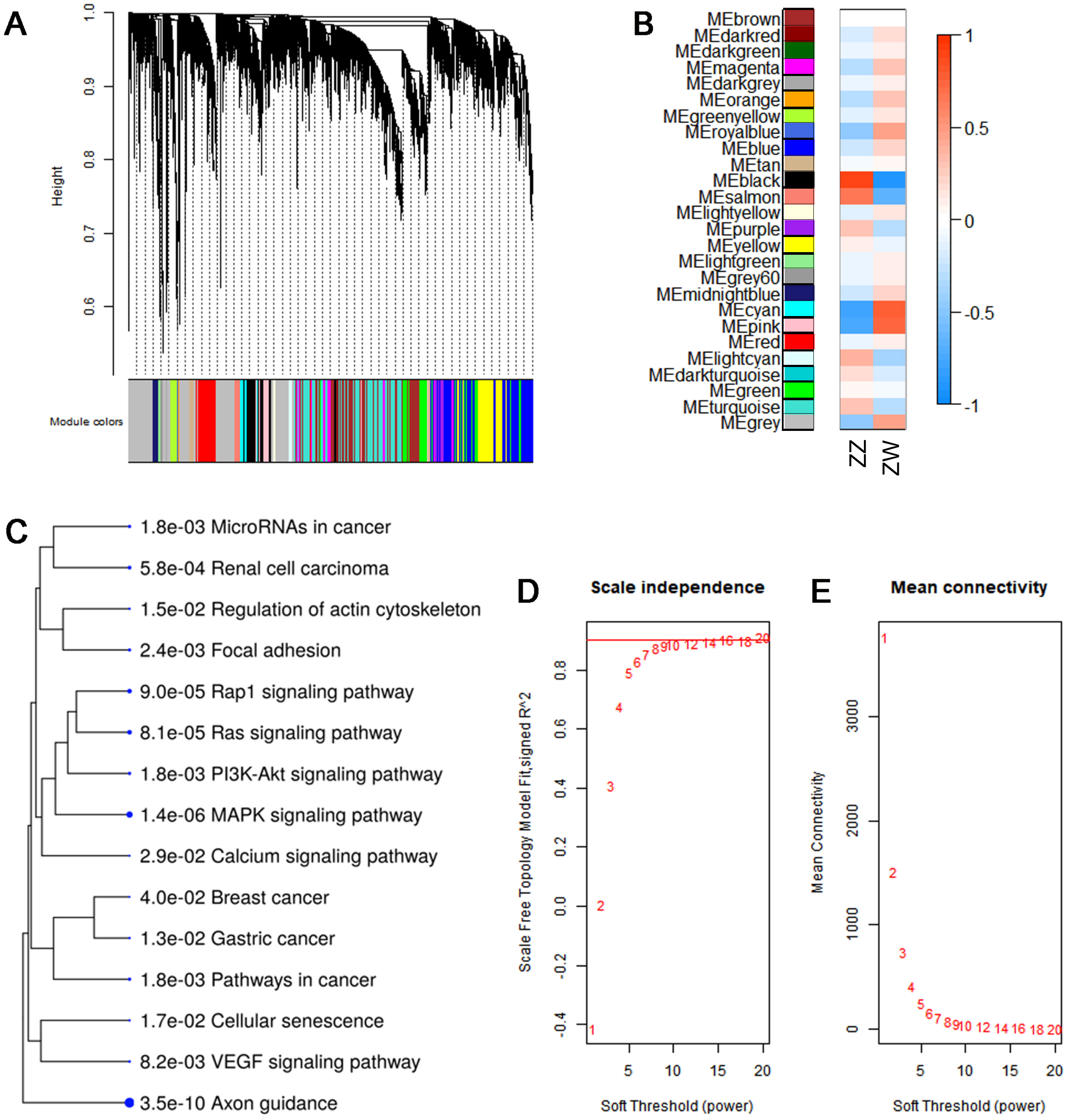

A
Height
Module colors
B
MEbrown
MEdarkred
MEdarkgreen
MEmagenta
MEdarkgrey
MEorange
MEgreenyellow
MEroyalblue
MEblue
MEtan
MEblack
MEsalmon
MElightyellow
MEpurple
MEyellow
MElightgreen
MEgrey60
MEmidnightblue
MEcyan
MEpink
MEred
MElightcyan
MEdarkturquoise
MEgreen
MEturquoise
MEgrey
ZZ
ZW
C
1.8e-03 MicroRNAs in cancer
5.8e-04 Renal cell carcinoma
1.5e-02 Regulation of actin cytoskeleton
2.4e-03 Focal adhesion
9.0e-05 Rap1 signaling pathway
8.1e-05 Ras signaling pathway
1.8e-03 PI3K-Akt signaling pathway
1.4e-06 MAPK signaling pathway
2.9e-02 Calcium signaling pathway
4.0e-02 Breast cancer
1.3e-02 Gastric cancer
1.8e-03 Pathways in cancer
1.7e-02 Cellular senescence
8.2e-03 VEGF signaling pathway
3.5e-10 Axon guidance
D
Scale independence
Scale Free Topology Model Fit,signed R^2
Soft Threshold (power)
E
Mean connectivity
Mean Connectivity
Soft Threshold (power)

Figure S7

A

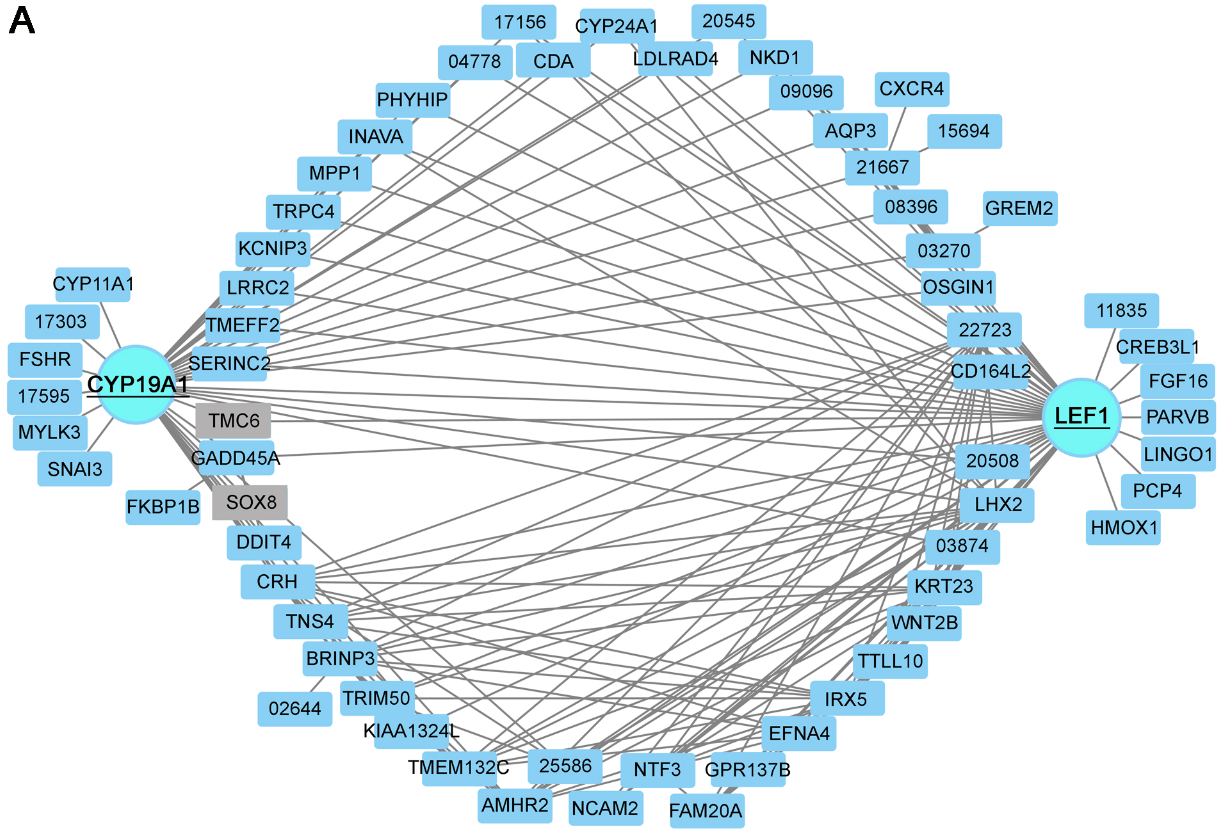
17156
CYP24A1
20545
04778
CDA
LDLRAD4
NKD1
PHYHIP
09096
CXCR4
INAVA
AQP3
15694
MPP1
21667
TRPC4
08396
GREM2
KCNIP3
03270
CYP11A1
LRRC2
OSGIN1
17303
TMEFF2
22723
11835
FSHR
SERINC2
CD164L2
CREB3L1
CYP19A1
FGF16
17595
TMC6
LEF1
PARVB
MYLK3
GADD45A
20508
LINGO1
SNAI3
PCP4
FKBP1B
SOX8
LHX2
HMOX1
DDIT4
03874
CRH
KRT23
TNS4
WNT2B
BRINP3
TTLL10
02644
TRIM50
IRX5
KIAA1324L
EFNA4
TMEM132C
25586
NTF3
GPR137B
AMHR2
NCAM2
FAM20A

B

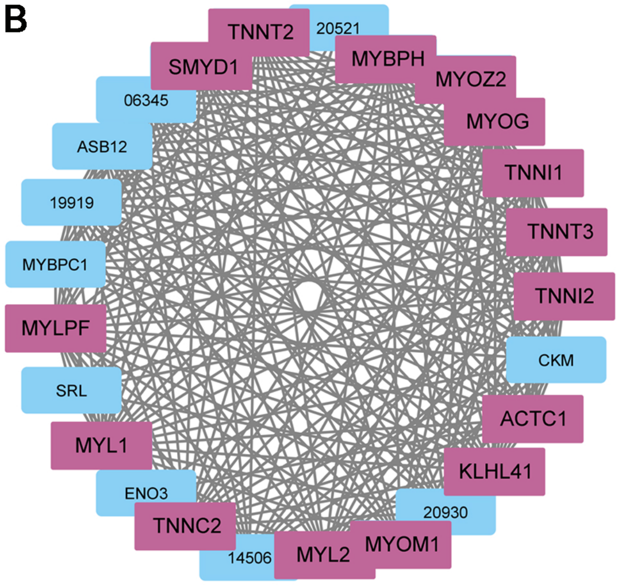
TNNT2
20521
SMYD1
MYBPH
MYOZ2
06345
MYOG
ASB12
TNNI1
19919
TNNT3
MYBPC1
TNNI2
MYLPF
CKM
SRL
ACTC1
MYL1
KLHL41
ENO3
20930
TNNC2
14506
MYL2
MYOM1

Figure S8

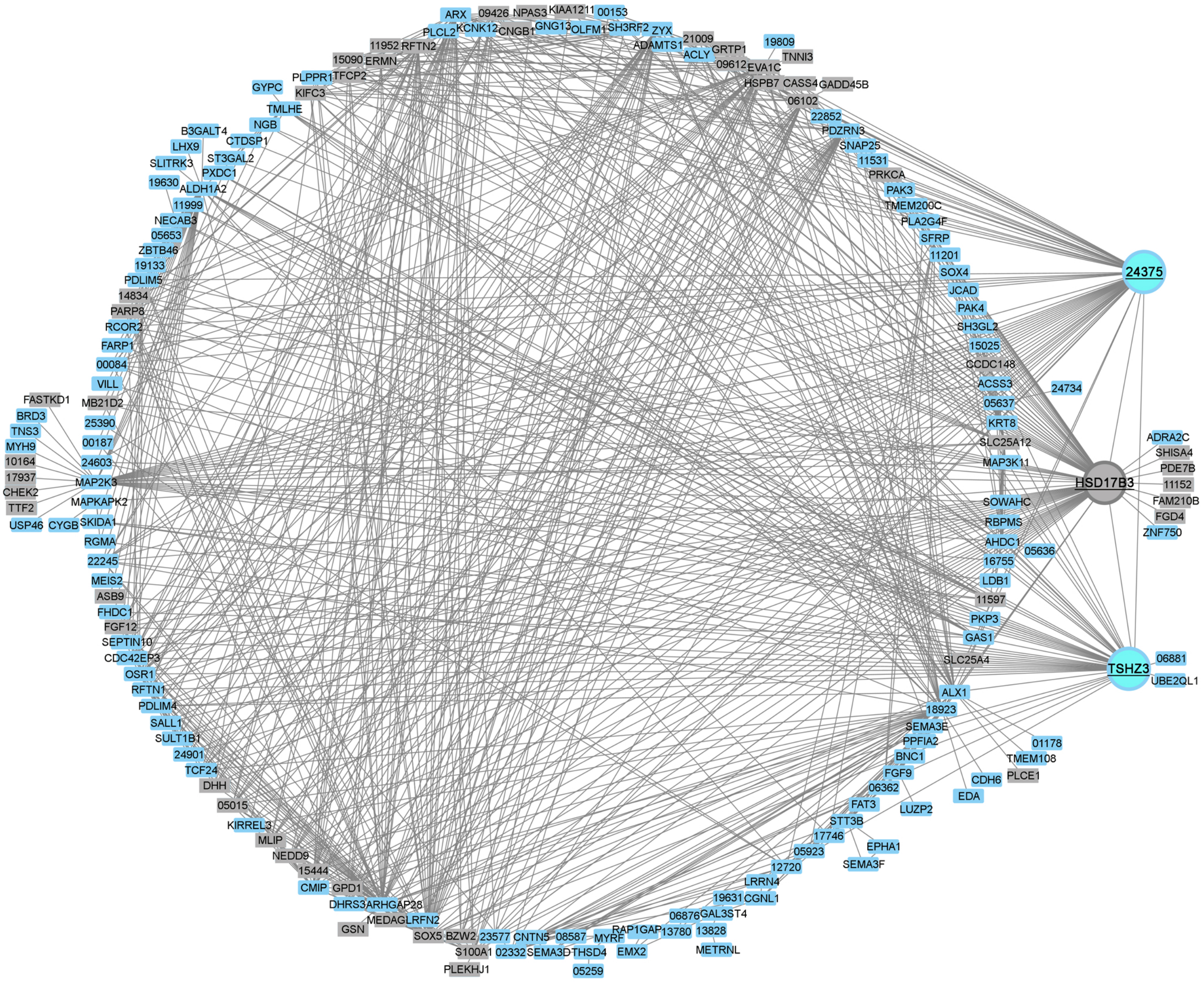

ARX
09426
NPAS3
KIAA1211
00153
PLCL2
KCNK12
CNGB1
GNG13
OLFM1
SH3RF2
ZYX
21009
19809
11952
RFTN2
ADAMTS1
GRTP1
15090
ERMN
ACLY
09612
EVA1C
TNNI3
PLPPR1
TFCP2
HSPB7
CASS4
GADD45B
GYPC
KIFC3
06102
TMLHE
22852
NGB
PDZRN3
B3GALT4
CTDSP1
SNAP25
LHX9
ST3GAL2
11531
SLITRK3
PXDC1
PRKCA
19630
ALDH1A2
PAK3
11999
TMEM200C
NECAB3
PLA2G4F
05653
SFRP
ZBTB46
11201
19133
SOX4
24375
PDLIM5
JCAD
14834
PAK4
PARP8
SH3GL2
RCOR2
15025
FARP1
CCDC148
00084
ACSS3
24734
VILL
05637
FASTKD1
MB21D2
KRT8
BRD3
25390
SLC25A12
ADRA2C
TNS3
00187
SHISA4
MYH9
24603
MAP3K11
PDE7B
10164
HSD17B3
11152
17937
MAP2K3
SOWAHC
FAM210B
CHEK2
MAPKAPK2
RBPMS
FGD4
TTF2
SKIDA1
AHDC1
ZNF750
USP46
CYGB
05636
RGMA
16755
22245
LDB1
MEIS2
11597
ASB9
PKP3
FHDC1
GAS1
FGF12
06881
SEPTIN10
TSHZ3
CDC42EP3
SLC25A4
UBE2QL1
OSR1
ALX1
RFTN1
18923
PDLIM4
SEMA3E
SALL1
PPFIA2
01178
SULT1B1
BNC1
TMEM108
24901
FGF9
CDH6
PLCE1
TCF24
06362
EDA
DHH
FAT3
LUZP2
05015
STT3B
KIRREL3
17746
EPHA1
MLIP
05923
SEMA3F
NEDD9
12720
15444
LRRN4
CMIP
GPD1
19631
CGNL1
DHRS3
ARHGAP28
06876
GAL3ST4
MEDAG
LRFN2
RAP1GAP
13780
13828
GSN
SOX5
BZW2
23577
CNTN5
08587
MYRF
S100A1
02332
SEMA3D
THSD4
EMX2
METRNL
PLEKHJ1
05259

Figure S9

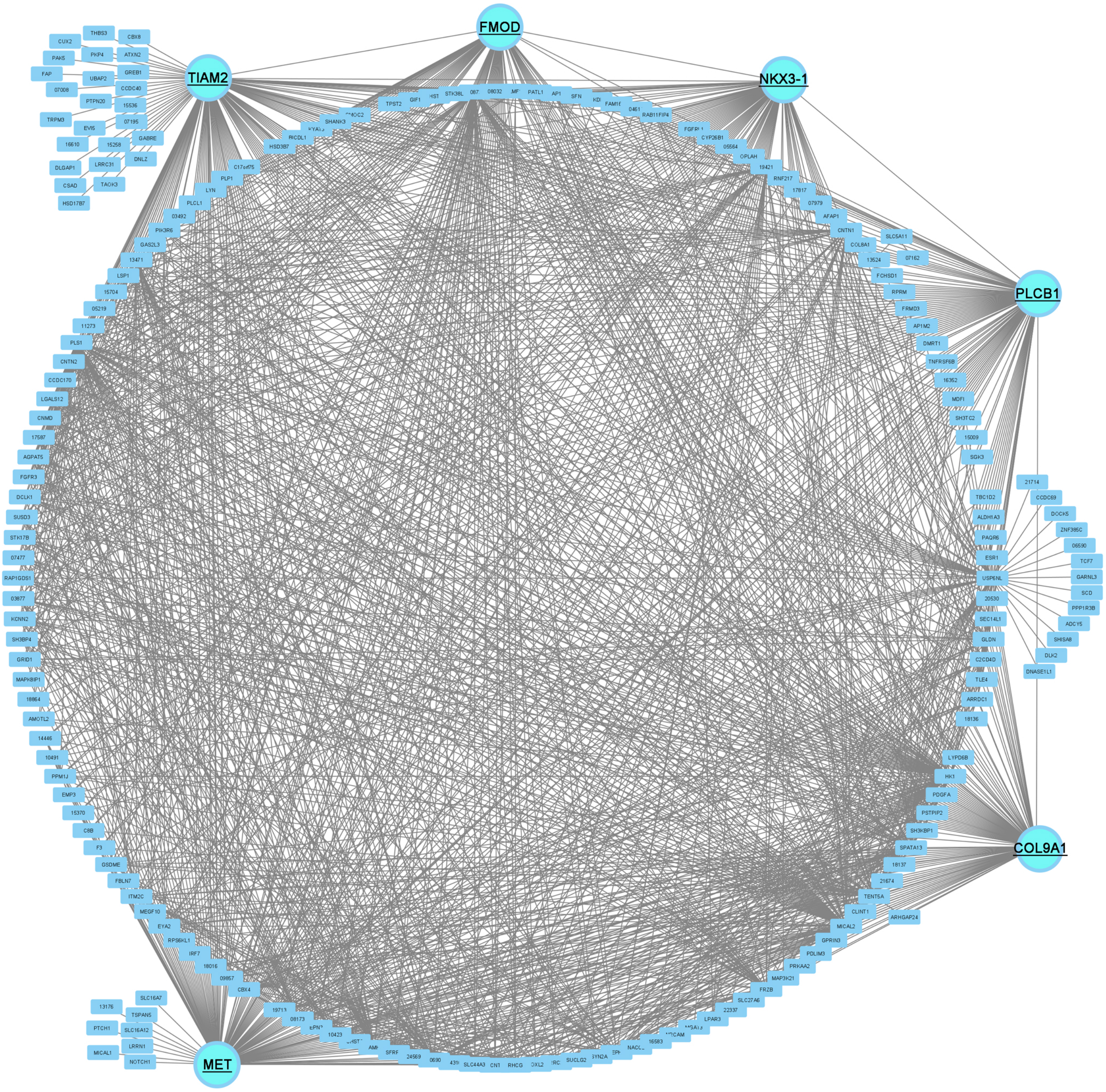

Figure S10

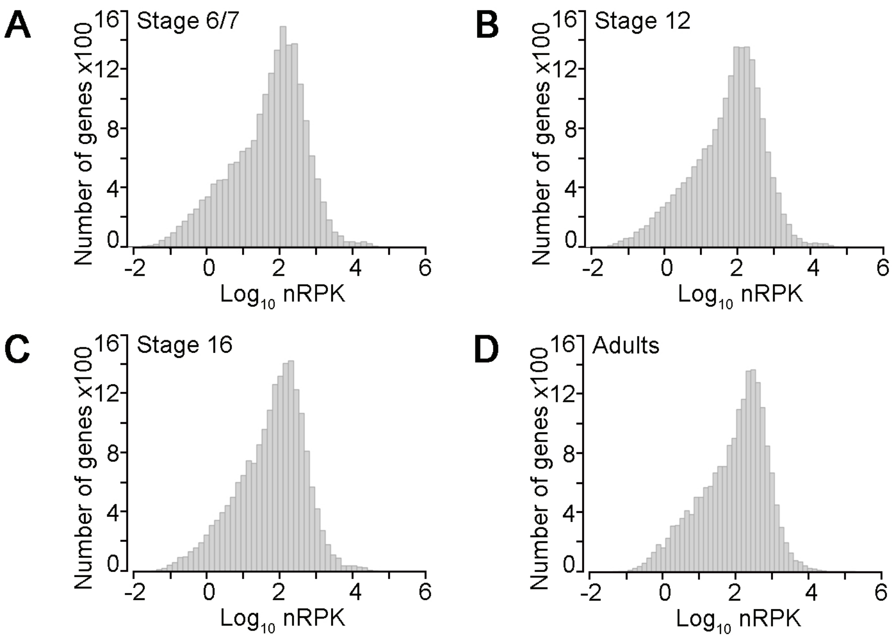

A
Stage 6/7
B
Stage 12
C
Stage 16
D
Adults
Number of genes x100
Log10 nRPK
